# Identification and statistical optimization of a novel alginate polymer extracted from newly isolated *Synechocystis algini* MNE ON864447 with antibacterial activity

**DOI:** 10.1186/s12934-023-02240-w

**Published:** 2023-11-07

**Authors:** Mabroka H. Saad, Nagwa M. Sidkey, Esmail M. El-Fakharany

**Affiliations:** 1 Protein Research Department, Genetic Engineering and Biotechnology Research Institute (GEBRI), City of Scientific Research andTechnological Applications (SRTA-City), New Borg AL Arab, Alexandria Egypt; 2 Botany & Microbiology Department, Faculty of Science, Al-Azhar University (Girls Branch), Cairo, Egypt; 3 Pharmaceutical and Fermentation Industries Development Centre (PFIDC), City of Scientific Research and Technological Applications (SRTA-City), New Borg Al-Arab, Alexandria Egypt

**Keywords:** Cyanobacterial alginate, Biopolymer, Statistical optimization, *Synechocystis algini*, Antibacterial activity

## Abstract

Cyanobacteria are a potential source of promising secondary metabolites with different biological activities, including antibacterial, antiviral, antifungal, antiprotozoal, and anticancer activities. To combat the emergence of antibiotic resistance, there is an urgent requirement for new drugs, and cyanobacteria metabolites can constitute alternative new antibacterial medication. The chemical complexity of their exopolysaccharides indicates that they have the potential to be bioactive molecules with many biological activities. The present study aimed to produce and optimise a novel alginate polymer from a newly isolated cyanobacterium, *S. algini* MNE ON864447, in addition to its promising antibacterial activity. We successfully isolated a new cyanobacterium strain, *S. algini* MNE ON864447 from the Nile River, which produces alginate as an extracellular polymeric substance. The isolated cyanobacterial alginate was identified using a set of tests, including FTIR, TLC, HPLC, GC–MS, and ^1^H NMR. Plackett–Burman statistical design showed that working volume (X1), the incubation period (X2), and inoculum size (X3) are the most significant variables affecting the production of alginate. The highest alginate production (3.57 g/L) was obtained using 4% inoculum size in 400 mL medium/L conical flask after 20 days of the incubation period. The extracted alginate showed potent antibacterial activity against both Gram-negative and Gram-positive bacteria and *Streptococcus mutants* (NCTC10449) are the most sensitive tested pathogen for purified cyanobacterial alginate with inhibition zone diameters of 34 ± 0.1 mm at 10 mg/mL of purified alginate while *Vibro cholera* (NCTC 8021) the lowest sensitive one and showed inhibition zone diameters of 22.5 ± 0.05 mm at the same cyanobacterial alginate concentration. This antibacterial activity is a critical step in the development of antibacterial drugs and presents a new challenge to fight against multi-resistant bacteria.

**Supplementary Information:**

The online version contains supplementary material available at 10.1186/s12934-023-02240-w.

## Introduction

Biopolymers are macromolecules derived from natural resources with several advantages including, biodegradability, biocompatibility, flexibility, non-toxicity, and their abundance in living organisms, making them a sustainable alternative to petroleum-derived such as poly (3-hydroxybutyrate) which is a promising polymer substituting petroleum plastics in several industrial uses [1–3]. The largest proportion of the global polysaccharide market is made up of polymers extracted from plants (e.g., starch, cellulose, and pectins), animals (hyaluronic acid, heparin, and chondroitin sulfate), and algae (alginate, carrageenan, and agar), while bacterial polysaccharides still account for a small fraction of the global market [4]. However, bacterial polysaccharides still attracted the attention of many scientists due to their unique advantages including, their fast growth rate, reduced polymer production times, ease of extraction, and the fact that they can be developed under controlled conditions to produce polymers with specific features, while algal and plant polysaccharides are greatly influenced by environmental conditions [5].

Cyanobacteria are considered an excellent source for polysaccharide production since most of the strains produce extracellular polymeric substances (EPS). The cyanobacterial EPS can be attached to the cell surface as a sheath, capsule, or slime, being designated as bound polysaccharides (BPs), or be released into the surroundings, being designated as released polysaccharides (RPs) [6, 7]. Cyanobacterial EPS displays a variety of functions, including gliding motility, structural, adhesive, and nutrient repositories in phototrophic biofilms, and protection against abiotic stress [8, 9]. Examples of the most common EPS extracted from cyanobacteria are sacran from *Aphanothece sacrum*, spirulan from *Arthrospira platensis*, and cyanoflan from *Cyanothece* sp. CCY 0110 [10–12]. Unlike other bacteria's polymers, which typically comprise four or fewer monomers, cyanobacterial polymers are heteropolysaccharides formed of a larger number of different monosaccharides (up to 13). Furthermore, the main characteristic features of cyanobacterial polysaccharides that make them very applicable compounds in biotechnological and biomedical fields are their amphiphilic nature, presence of sulfate groups, strong anionic nature, and wide range of potential structural configurations [13–15]. Moreover, their photoautotrophic metabolism eliminates the need for carbon feedstocks and reduces high-cost production. To increase production and create structural and compositional variants that are particularly tailored for a specific application, a greater understanding of the cyanobacterial EPS biosynthesis pathways is required.

Antimicrobial resistance is one of the world's major causes of mortality. Recent statistics suggest that 1.2 million fatalities directly resulted from resistant bacterial infections in 2019, and 4.95 million deaths were related to resistant bacterial infections. The increase of multidrug-resistant Gram-negative bacteria, especially carbapenemase-producing *Enterobacteriaceae*, methicillin–resistant *Staphylococcus aureus* (MRSA), vancomycin–resistant *Enterococcus*, and multi–drug–resistant *Mycobacterium tuberculosis*, is alarming. Gram-negative and Gram-positive resistant bacterial strains cause a variety of hospital-acquired and community-acquired infectious diseases, including wound infections, osteomyelitis, surgery-associated and implant-associated infections, and respiratory infections [16]. As a result, there is an urgent need for medications that control and avoid microbial resistance, whether in the form of novel antibiotics or antimicrobial agents. Although, several studies have displayed the activities of cyanobacterial EPS as anticoagulant, antioxidant, immunomodulatory, anticancer, and antiviral drugs in biomedical applications [17], the publications concerned with their antibacterial activity are few. The antimicrobial activity of cyanobacterial EPS is also reported in the literature. EPS extracted from *Gloecapsa* sp. Gacheva 2007/R-06/1 and *Synechocystis* sp. R10 exhibited antimicrobial activity against a wide range of food-borne pathogens [18]. *Arthrospira platensis* EPS also exhibited antimicrobial activity against both Gram-negative and Gram-positive bacteria [19]. Although, structural information about cyanobacterial EPS is scarce, their biological activities are significantly related to the presence of sulfate groups and negative charge [7].

Despite all the published studies that discussed different alginate applications in various life aspects, those concerned with antibacterial activity are few and restricted to few bacterial pathogens [20]. This study aimed to investigate and optimize the production of cyanobacterial alginate for the first time from a newly cyanobacterium isolate; *S. algini* MNE ON864447, and the characterization of this polymer using FT-IR, GC–MS, HPLC, and 2D-NMR. In addition, examine their antibacterial activity against a wide range of Gram-negative and Gram-positive bacterial pathogens as the initial step to studying their biological activity. We are looking forward to further studies about cyanobacterial alginate structure (its building block distribution) and its exact antibacterial mechanism.

## Materials and methods

### Isolation and purification of different cyanobacterial isolates

#### Samples collection method and cyanobacteria isolation from different soil and water samples

Random soil and water samples were collected from various Egyptian governorates, including (Beheira governorate (DMS, 30° 36′ 36″ N, 30 25′ 48″ E), Monufia governorate (DMS, 30° 31′ 12″ N, 30°59′ 24″ E), Alexandria governorate (DMS, 31°10′ 0″ N, 29° 53′ 0″ E), and Gharbia governorate (DMS, 30° 52′ 1.2″ N, 31° 1′ 40.8″ E). Soil samples were collected from 5 to 10 cm-deep soil layers and stored in tightly sealed plastic bags. Soil samples were sieved, dried, and stored in wide mouth screw cap glass bottles. While solid rocks were scratched to obtain fresh biomass, which was then transported to the lab for cyanobacterial isolation and stored in glass bottles and tightly sealed plastic bags. Vegetative growth of cyanobacteria in aquatic samples was collected in 200 mL sterile jars containing at least 20 mL sterile enriched Bold's Basal Medium (BB) which consists of NaNO_3_ (2.94 mM), CaCl_2_.2H_2_O (0.17 mM), MgSO_4_.7H_2_O (0.30 mM), K_2_HPO_4_ (0.43 mM), KH_2_PO_4_ (1.29 mM), NaCl (0.43 mM), 0.05 mL of EDTA stock (50 g of EDTA acid form, and 31 g of KOH were dissolved in 1L of H_2_O and stored at room temperature in darkness), 0.05 mL Iron stock (10 mL of conc H_2_SO_4_ mixed with 5 g of FeSO_4_.7H_2_O, bring the total volume to 1L and stored at room temperature in darkness.), 0.05 mL Boron stock (11 g of H_3_BO_3_ was dissolved in 1L H_2_O and stored at room temperature in darkness.), and 0.05 mL Bold trace stock (10 mL/L of conc H_2_SO_4_, ZnSO_4_.7H_2_O (1.50 µM), MnCl.4H_2_O (0.36 µM), MoO_3_ (0.26 µM), CuSO_4_.5H_2_O (0.31 µM), and Co(NO_3_)_2_.6H_2_O (0.084 µM) were dissolved in 1L H_2_O and stored at room temperature in darkness). To optimize in vitro cyanobacterial growth, water body parameters such as pH and temperature should be recorded [21]. Soil samples were dried in an oven at 60 °C for 15 min before sieving to remove ambigous particles. A small amount (1 g) of sieved soil was applied in the middle of the sterilized plates of Z^8^ semi-solid medium (MgSO_4_·7H_2_O (0.25 g), NaNO_3_ (0.467 g), Ca(NO_3_)_2_·4H_2_O (59 mg), NH_4_Cl (31 mg), Na_2_CO_3_ (0.02 g), Fe-EDTA solution (10 ml), 1 ml of Gaffron micronutrients [(H_3_BO_3_(3.1 g), MnSO_4_·4H_2_O (2.23 g), ZnSO_4_·7H_2_O (0.22 g), (NH_4_)_6_Mo_7_O_24_·4H_2_O (0.088 g), Co(NO_3_)_2_·6H_2_O (0.146 g), VOSO_4_·6H_2_O (0.054 g), Al_2_(SO_4_)_3_K_2_SO_4_·2H_2_O (0.474 g), NiSO_4_(NH_4_)_2_SO_4_·6H_2_O (0.198 g), Cd(NO_3_)_2_·4H_2_O(0.154 g), Cr(NO_3_)_3_·7H_2_O(0.037 g), Na_2_WO_4_·2H_2_O (0.033 g), KBr (0.119 g), KI (0.083 g), deionized water to1 L], and 0.8 g/L agarose separately autoclaved and used for medium solidification) [22], which was supplemented with 0.4 g/L sodium sulfite [23]. Penicillin and cycloheximide were also added at a final concentration of 100 µg/mL. The plates were then sealed with parafilm and incubated at 27 ± 3 °C with continuous light at 1200 LUX. Every day, the plates were checked for the presence of any cyanobacterial growth [24, 25]. Cyanobacteria isolation from liquid samples is based on the number of cyanobacteria in water samples. Using a light microscope (Binocular NSL—CX23 Olympus Optical Co., LTD. Japan), samples with minimal cyanobacterial growth can be concentrated and cultured in an enriched liquid medium, while those with abundant growth can be diluted and subjected to the following purification processes.

### Cyanobacteria purification methods

Different microbiological purification techniques were combined to get axenic cyanobacterial isolates, including the density centrifugation technique using Z^8^ liquid medium flasks in triplicates and incubated with continuous illumination at 1200 LUX. The incubated flasks at 27 ± 3.0 °C were examined daily for the presence of any cyanobacterial growth. A serial dilution of collected samples from 10^–1^ to 10^–10^ was prepared, and 50 µl of each diluted sample was spread on Z^8^ semi-solid medium. Then plates were incubated at 27 ± 3.0 °C with continuous illumination at 1200 LUX using white fluorescence lamps for 3–4 weeks [26, 27]. In addition, micromanipulation techniques using an inverted microscope and sterile pasteur pipette were used to pick up pure colonies. The incubated plates were examined daily for any early growing filament or cyanobacterial colonies under a light microscope, and any single separate colony was picked up using a micropipette and transferred to 10 mL sterile medium followed by vortex and centrifuged at 805 xg (Eppendorf 5810R Refrigerated Centrifuge with an 18 cm rotor radius). The supernatant was discarded, and the pellet was washed several times by repeated centrifugation at 805 xg using 1% (v/v) kanamycin solution. Finally, pellets were suspended and spread with a needle on Z^8^ semi-solid plates and incubated at 27 ± 3.0 °C with continuous illumination at 1200 LUX for 3–4 weeks [28].

### Polysaccharides extraction and selection of the most polysaccharide-producible isolate

#### Method for bounded polysaccharide (BPS) extraction

The different cyanobacterial isolates were grown in 500 mL Erlenmeyer flasks containing 300 mL of a modified BG11 medium [29] consisting of the following components (g/L): NaNO_3_, 1.5; K_2_HPO_4_, 0.04; MgSO_4_.7H_2_O, 0.075; CaCl_2_.2H_2_O, 0.036; citric acid, 0.006; disodic EDTA, 0.001; NaHCO_3_, 1.7; ammonium iron citrate, 0.006; NaCl, 10,000 and 1 mL of metal salt solution [H_3_BO_3_(2.86 g), MnCl_2_.4H_2_O (1.81 g), ZnSO_4_.7H_2_O (0.222 g), Na_2_MoO_4_.2H_2_O (0.039 g), CuSO_4_.5H_2_O (0.079 g), Co(NO_3_)_2_.6H_2_O (0.0494 mg) up to 1 L deionized water]. Then 5 mL of each isolate suspension (2 × 10^6^ cells/mL) was used as inocula and flasks were incubated as usual at 27 ± 3.0 °C under a continuous light intensity of 1200 LUX. Cyanobacterial cultures were filtered using Whatman GF/A filter paper and the biomass was washed three times with distilled water, filtrated, and dried in an oven at 45 °C for 12 h before being crushed into powder. To deactivate endogenous enzymes and remove colored components and tiny molecular contaminants, the dried powder was defatted with 80% ethanol for 2 h at 80 °C. The mixture was then allowed to cool at ambient temperature before being filtered again using Whatman GF/A filter paper. The resultant biomass was subjected to the hot water extraction technique [30]. In brief, 5 g of dried and defatted biomass was extracted using a predetermined extraction ratio of deionized water to defatted biomass (10–30, V/W), extraction temperature (70–100 °C), and extraction duration (3–4 h). After three rounds of extraction, the combined extracts were centrifuged at 3000 xg for 15 min. The supernatant was collected, concentrated, and precipitated in three equal volumes of 99% ethanol for 24 h at 4 °C. The precipitate was deproteinized with sevag solution (chloroform:butyl alcohol, 4:1), recovered by centrifugation at 3000 × g for 20 min., and lyophilized in order to get pure polysaccharide. The extraction yield was calculated as the difference between the dry weight of the extracted crude polysaccharides (W1) and the initial weight of the cyanobacterial isolate (W0) [31]: Yield of polysaccharides (%) = (W1/W0)*100.

#### Method for extracellular polysaccharide (EPS) extraction

At the end of the stationary phase, cyanobacterial biomass was harvested from the growing medium using Whatman No. 1 filter paper. The culture filtrate was centrifuged at 3220 xg for 20 min at 4 °C and three equal volumes of absolute ethanol were added to the cell-free filtrates and stored at 4 °C for 24 h for polymer precipitation. After incubation, it was centrifuged at 3220 xg for 20 min at 4 °C [8]. The resulting compound was subsequently lyophilized, and the polysaccharide yield for each culture was calculated as previously mentioned. The carbohydrate content of the purified polysaccharide was measured according to Dubois et al. [32] using the phenol sulphuric acid method. A 0.5 mL of purified polysaccharide solution (0.1 g/ mL) was vortexed with a 0.5 mL of 5% phenol solution. Then, the mixture was vortexed with 2.5 mL of concentrated sulphuric acid and left to stand for 30 min at 37 °C. The carbohydrate amount was determined by reading absorbance at 490 nm using glucose at different concentrations to create a calibration curve.

### Identification of the most polysaccharide-producible isolate

#### Morphological characterization and cultivation condition investigation

A morphological study of axenic cyanobacterial isolate under light microscopy and their identification were carried out using the morphological keys of Desikachary [33]**.** For scanning electron microscopy (SEM), the cultures were dried and coated with gold. The gold-coated specimen was examined at different magnifications with Analytical Scanning Electron Microscope (Jeol JSM-6360 LA operating at 20 kV). The effects of different pH values (2–10), temperature (10–80 °C), and NaCl concentrations (0, 10, and 20 g/L), on the growth rate of the selected isolate were studied in BG11 medium. For heterotrophic cultivation, 5 mL of cell suspension (2 × 10^6^ cells/mL) was washed twice with distilled water and inoculated in 100 mL BG11 medium supplemented with 20 g/L of glucose and yeast extract and incubated in the dark shaker at 100 rpm and 27 ± 3.0 °C. The cultures were harvested and dry cyanobacterial biomasses were determined.

### 16S rRNA analysis

DNA extraction from cyanobacteria isolates was performed according to Nübel et al. [34]. Cyanobacterial 16S rRNA was amplified through polymerase chain reaction (PCR) with specific primers; CYA106F (CGG ACG GGT GAG TAA CGC GTG A), CYA781R (a) (GAC TAC TGG GGT ATC TAA TCC CAT T) and CYA781R (b) (GAC TAC AGG GGT ATC TAA TCC CTT T). Sequencing of obtained 16S rRNA gene was performed by Bio vision Company and submitted to the NCBI GenBank database through the Submission Portal tool (https://submit.ncbi.nlm.nih.gov/subs/genebank/). Then, our sequence was aligned and compared with other deposited organism sequences in the same species available in the genebank database (htt://www.ncbi.nlm.nih.gov) and the neighbor-joining phylogenetic tree was created using MEGA11 software.

### Physicochemical properties of extracted polysaccharide

The pH was determined using the AOAC (Association of Official Analytical Chemists, 1884). The purified polysaccharide (1 g) was dissolved in 100 mL of distilled water, and the pH was measured at room temperature with a Jenway portable pH meter (Model 550). The color was visually inspected on a background of white and dark lighting. The polysaccharide moisture was evaluated according to AOAC (Association of Official Analytical Chemists, 1884) by measuring the mass loss of purified polysaccharide after 24 h of heating at 105 ± 1 °C. In addition, the harvested cyanobacterial polysaccharide was combined with ion bond-forming chemicals such as K_2_Cr_2_O_7_, ZnSO_4_, CuSo_4_, and KMnO_4_ to investigate the precipitation or gel formation property [35]. About, 250 µl of NaOH (30%) was combined with 5 ml of aforementioned ionic solutions (0.16%), then 500 µl of cyanobacterial polysaccharide (0.6%) was added and the combination was tested for gel-forming ability.

### Purity evaluation of extracted polysaccharide and its biochemical analysis

The presence of alkaloids, flavonoids, tannins, phlorotannins, terpenoids, steroids, saponins, phenols, and glycosides was analyzed to determine the polysaccharide purity by Evans [36], which are quick qualitative tests to confirm the presence of contaminants (other cyanobacterial metabolites accompanied by targeted compounds). The presence of total flavonoids was tested according to the methods of Sakanaka et al. [37]**,** alkaloids were determined by Mayer’s Test [38] and the presence of tannins was determined using a ferric chloride test [39]. In addition, a qualitative approach was utilized to assess the presence of terpenoids and steroids according to protocol of Kumar [40]. While total phenolic compounds were tested using the protocol of González et al. [41]. The carbohydrate types of the extracted polysaccharide were examined using standard techniques such as the Molisch's test, the Iodine test, the carbazole test, the Seliwanoff test, and the Osazone test [42, 43]. However, the total proteins were evaluated as described by the Bradford method [44] using BSA as a standard protein and the protein was determined as %DW. The total lipids and Ash were gravimetrically determined using chloroform–methanol following AOAC (1884) protocols.

### Characterization of purified polysaccharide from the selected isolate

#### Thin layer chromatography (TLC)

The extracted cyanobacterial exopolysaccharide were hydrolyzed in 5 mL of sulfuric acid for 6 h and visualized using the TLC technique. The location of the tested samples and standard alginate on the plate is numerically represented by calculating a retention factor (Rf).

#### High-performance liquid chromatography (HPLC)

The extracted polysaccharides (1 mg/mL) were dissolved in high pure distilled water and subjected to HPLC quantitative and qualtitative analysis. The HPLC (Agilent 1000) analysis of extracted polysaccharide and standard alginate was separately performed on the Aminex HPX-42A column (7.8 × 300 mm I.D). The column was operated with a flow rate of 0.5 mL/min and the column temperature was kept at 85 °C. The refractive index detector was used to detect the output signals at 280 nm.

#### Examining monosaccharide composition using gas chromatography–mass spectroscopy (GC–MS)

The purified polysaccharide was subjected to acid hydrolysis and derivatization for sugar composition determination. Then, derivatized sugars were determined using a GC-TSQ mass spectrometer (Thermo Scientific, Austin, TX, USA) with a direct capillary column TG–5MS (30 m × 0.25 mm × 0.25 µm film thickness). The column oven temperature was initially held at 60 °C and then increased by 6 °C/min to 250 °C with held for 1 min, then increased to 300 at 30 °C/min. Helium was used as a carrier gas at a constant flow rate of 1 ml/min. The solvent delay was 4 min and diluted samples of 1 µl were injected automatically using Autosampler AS3000 coupled with GC in the split mode. Electron ionization (EI) mass spectra were collected at 70 eV ionization voltages over the range of m/z 50–650 in full scan mode. The ion source and transfer line were set at 200 °C and 280 °C, respectively. The components were identified by comparison of their mass spectra with those of WILEY 09 and NIST14 mass spectral databases [45].

### Characterization of the purified polysaccharide using FTIR analysis

The Fourier Transform Infrared Spectroscopy (FTIR) approach was employed to determine the functional groups of the purified polysaccharide. The dried polysaccharide was crushed into a fine powder (1 mm) and mixed with dried KBr powder to form a homogenous compressed thin tablet, which was then placed in a sample pan and the infrared spectrum was recorded with a Bruker Alpha FTIR spectrometer. The spectra were all the average of two independent observations from 3500 to 500 cm^−1^ taken across 128 scans at a resolution of 2 cm^−1^ [46].

### Characterization of the purified polysaccharide using NMR analysis

The expected structure for the purified polysaccharide was confirmed using one and two-dimensional NMR spectra. All two-dimensional experiments were acquired using a pulse field gradient incorporated into NMR pulse sequences. The two-dimensional homonuclear ^1^H-^1^H correlation spectroscopy (COSY) was obtained with 128 2040 data points with a spectral width of 1200 Hz and processed in a 1024 × 1024 matrix to achieve a final resolution in the two dimensions close to 2.3 Hz/point. The 2D^1^H-^13^C HSQC spectra were obtained with 128 × 1024 data points and processed in a 1024 × 1024 matrix to yield a final resolution near 2.3 Hz/point in ^1^H and close to 2.4 Hz/point in ^13^C. The number of scans (ns) in each experiment was determined by the concentrations of the samples. TOPSPIN (3.5) software was used to analyze the spectra.

#### Fractionation of cyanobacterial alginate (MM, GG, and MG block determination)

The uronic acid blocks of cyanobacterial alginate were determined using the procedure of Lee et al. [47] with some modifications. The suspension of the partial hydrolyzed sample was allowed to cool at room temperature before being centrifuged at 3220 xg for 15 min. The phenol–sulphuric acid technique was used to estimate the quantity of MG blocks in the supernatant [32]. While the residue was suspended in 0.1 M sodium chloride and neutralized with 0.2 M sodium hydroxide to be dissolved. Then, the solution was adjusted with 0.1 M hydrochloric acid to attain a final pH of 2.8–3.0 for precipitation. The mixture was diluted to 10 mL with 0.1 M sodium chloride and centrifugation at 3220 xg for 15 min to obtain the precipitate. Both the precipitate and the supernatant were homopolymeric blocks of guluronic acid residues (GG blocks) and mannuronic acid (MM blocks), respectively. The quantities (% w/w) of MM and GG blocks were determined as compared to the alginate extracted from *Macrocystis pyrifera* with the M/G ratio of 1.56 (viscosity of 2% solution at 25° C approx. 14,000 cps, SIGMA^®^) using the phenol–sulphuric acid technique. The M/G ratio was estimated from MM and GG blocks, assuming the alternating MG blocks have an M/G ratio of 1.0 as reported by Haug et al. [48]. Based on the values of MG, MM, and GG block composition obtained from polymer fractionation, the formula used to determine the M/G ratio was (%MMx2 + %MG)/(%GGx2 + %MG).

### Statistical optimization of cyanobacterial polysaccharide production

#### Selection of the significant variables using Plackett–Burman design

The Plackett–Burman experimental design used in the current investigation included 20 experiments. was used to screen Seventeen independent factors for their impacts on the production of polysaccharides, including A (Sodium bicarbonate;g/L), B (sodium nitrate; g/L), C (dipotassium hydrogen phosphate; g/L), D (Magnesium sulfate heptahydrate; g/L), E (Calcium chloride; g/L), F (Citric acid; g/L), G (Ferric ammonium citrate; g/L), H (EDTA; g/L), J (Trace metal; mL), K (Sodium chloride; g/L), L (Temperature; °C), M (pH), N (working volume; mL), O (Inoculum size; %), P (Agitation speed; rpm), Q (Culture old; days), R (Incubation period; days). There are two levels for each variable, high ( +) and low ( −).

### Face-centered central composite design (FCCCD)

In the current study, three independent variables were examined at 20 tests at five different levels (−1.68, −1, 0, 1, 1.68) using a design that included 20 experiments. These variables are the working volume (X1), the incubation period (X2), and the inoculum size (X3). A total of 20 runs were carried out to optimize the levels and investigate the interactions between the selected parameters and cyanobacterial polysaccharide production.

### Studying the antibacterial activity of cyanobacterial polysaccharide

#### Preparation of different bacterial inocula

Gram-positive bacterial strains, including *Staphylococcus aureus* (ATCC25923), Methicillin resistance* Staphylococcus aureus* (MRSA), were obtained from Almery University Hospital (Alexandria, Egypt). Gram-negative bacteria strains, including *Klebsiella pneumonia* were obtained from Al-Azhar University Mycology Center (Cairo, Egypt).* Streptococcus mutants* (NCTC10449), *Bacillus subtilis* (NCTC 10400), *Vibro cholera* (NCTC 8021), *Salmonella typhi* (ATCC 19430), and *Escherichia coli* (ATCC 25922) were purchased from ATCC. All bacterial strains were inoculated in 20 mL of nutrient broth medium and incubated at 37 °C and 120 rpm overnight. Then, bacterial growth was measured at 600 nm using a microtiter plate reader (BIO-RAD, USA) and adjusted for the agar-well diffusion assay.

### Agar well diffusion assay

Agar well diffusion assay was achieved to evaluate the antibacterial activity of cyanobacterial exopolysaccharide as described by Chauhan et al. [49]. Briefly, molten cooled nutrient agar medium was poured into sterile Petri dishes at a height of 4 mm and left to solidify. After solidification, 0.5 ml of each standardized bacterium strain (10^5^ CFU/ml) was swabbed on the surface of agar plates. Then, a sterile 10 mm cork borer was used to make wells on each plate. Subsequently, cyanobacterial exopolysaccharide at different concentrations (10, 5, and 2.5 mg/mL) was poured into each well and allowed to diffuse for 3 h at 8 °C. The plates were examined for the presence of an inhibition zone after overnight incubation at 37 °C, and its diameter was measured in millimeters (mm) using a ruler.

### Statistical analysis

Data were expressed as ± SD using graph pad prism(version 9). The experimental design and statistical analysis were carried out using the Windows edition of Design-Expert software (version 13, Stat-Ease, Minneapolis, USA) (https://www.statease.com/sostware/design-expert/).

## Result and discussion

### Screening of cyanobacterial isolates for polysaccharide production

A total of 25 morphologically different cyanobacterial strains were screened for their potential polysaccharide production. All isolates were subjected to two extraction processes and the determination of total polysaccharide in 1 L culture medium. Our survey depends on the determination of the ability of isolates to produce bound (BPS) or free polysaccharides (FPS) under fermentation conditions of 120 rpm, 1200 LUX, and 30 °C for 30 days. The phenol–sulfuric acid method was used to determine the total polysaccharide content of 25 characterized cyanobacterial isolates. Additional file 1: Table S1 presents a list of our isolates; only six isolates (*Tetraedron* sp. Mab 6,* Chlorella* sp. mab 8, *Synechocystis* sp. Mab 16, *Chlorella* sp. Mab 18, *Synechocystis* sp. Mab 20, and *Synechococcus* sp. Mab 22) produced exopolysaccharides free in their culture media, while the other isolates retained their polysaccharides attached to their cells. Also, our survey revealed that five cyanobacterial isolates exhibited high polysaccharide yield; *Synechocystis* sp. (Mab 20, 1.2 g/L, 93.2% yield), *Tetraedron* sp. (Mab 6, 1.01 g/L, 93% yield), *Synechococcus* sp. (Mab 22, 0.91 g/ L, 88% yield), *Spirulina* sp. (Mab 10, 0.79 g/L, 77% yield), and *Nostoc* sp. (Mab 2, 0.72 g/L, 75% yield). The most potent microalgal isolate, *Synechocystis* sp*.* (strain Mab 20) was selected for further polysaccharide production and studies. It has been proposed that the production of exocellular polysaccharides in microorganisms, particularly cyanobacteria, serves an important function in shielding cells from stress and other harmful conditions. Several types of research have been conducted to investigate the capacity of various polysaccharide-producing cyanobacteria to escape stress caused by desiccation or low water activity in a desert or saline environment. Hill et al. [50] indicated that the released glycan offers storage for water, functions as a buffer between cells and the atmosphere, and represents a significant component of the process utilized by this cyanobacterium to resist desiccation. Moreover, it has been suggested that the polysaccharide's only function is to enhance motility by changing the surface characteristics of the trichome and/or the substrate. As previously seen in *Oscillatoria* sp., trichomes have the propensity to migrate back and forth within slack sheaths of slime, which serve as the trichome's actual paths of movement [51]. Consequently, numerous different forms of filamentous cyanobacteria appear to require polysaccharide slime to offer a favorable surface for movement.

### Morphological and phylogenetic characterization of the potent isolate

**Fig. 1:**
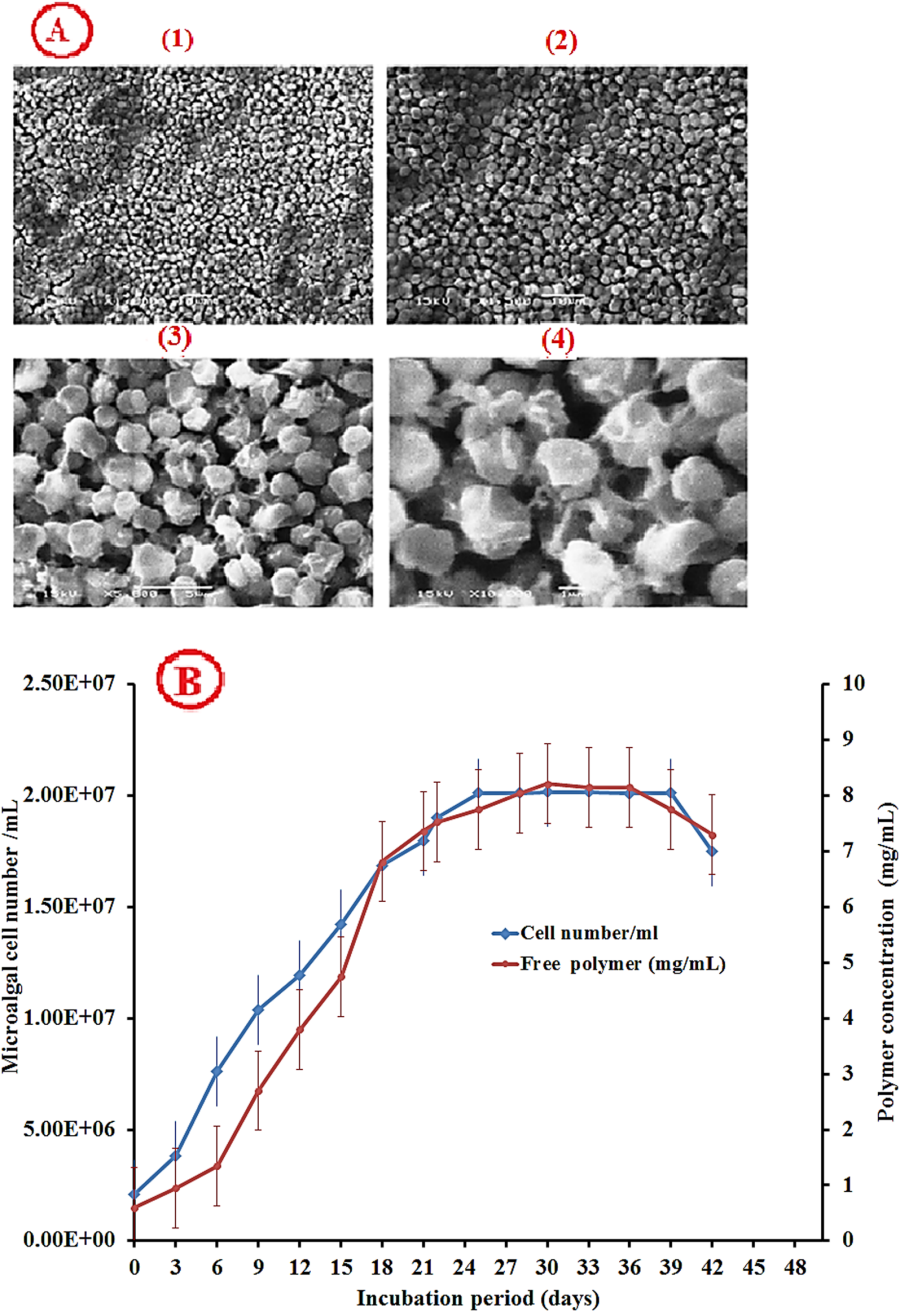
**A** Scanning electron microscopy showing cell morphology of *Synechocystis* sp. Mab 20 at a different magnification of 1000 X (1), 1500X (2), 5000X (3), and 10000X (4). **B** The growth curve of *Synechocystis* sp. Mab 20. and the produced polymer concentration at 30 °C and 1200 LUX for 40 days

**Fig. 2:**
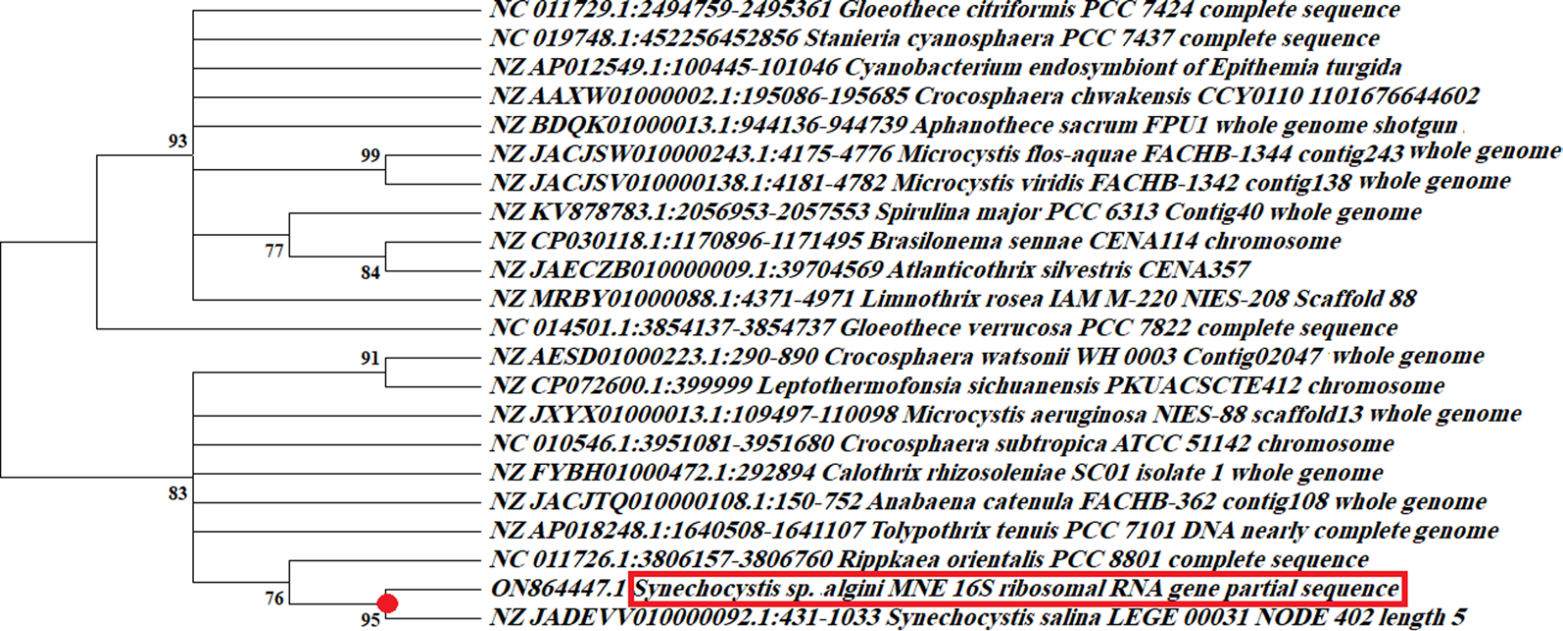
The phylogenetic tree of *S. algini MNE ON864447* was created by using software of MEGA version 11

Microscopic examination of the most potent isolate revealed that it is a freshwater, unicellular cyanobacteria and was classified as *Synechocystis* genus, *Merismopediaceae* family, *Synechococcales* order, *Cyanophyceae* class, *Cyanobacteria* phylum, and *Bacteria* domain (Fig. 1A). *Synechocystis* sp. strain Mab 20 displayed a typical growth curve with a clear exponential phase observed from day 3 to day 18 of culture time. Total EPS production by cyanobacterial isolates gradually increased during the exponential growth phase and maximized production was detected during the stationary phase. The maximum exopolysaccharide yield was 8.22 ± 0.014 mg/mL detected on 30^th^ of *Synechocystis* sp. culturing (Fig. 1B). Also, the cultivation condition displayed that *Synechocystis* sp. strain Mab 20 can grow only photo-autotrophically, not heterotrophically as it cannot utilize glucose as the main carbon source. Our strain is also able to tolerate high incubation temperatures (40 °C) and alkaline medium (pH = 10). Furthermore, *Synechocystis* sp. strain Mab 20 can survive under environmental stresses including salt stress (up to 10% NaCl) and cold stress (temperature = 14 °C) (data not shown). An axenic culture of *Synechocystis* sp*.* strain Mab 20 was identified by 16S rRNA sequencing. The obtained 16S rRNA fragment sequence was amplified by polymerase chain reaction (PCR) using specific sequencing primers, and the sequencing product was 800 bp. The obtained 16S rRNA gene sequence was aligned with the related 16S rRNA gene sequences available in the National Centre for Biotechnology Information (NCBI) databases using the BLASTN. The phylogenetic tree was generated using MEGA version 11 software using neighbor-joining analysis (Fig. 2). Only branches corresponding to bootstrap values above 50% of bootstrap replicates are expressed. Based on the 16S rRNA gene sequence, phylogenetic analysis revealed that *Synechocystis* sp. strain Mab 20 shared a high degree of similarity (98.51%) with *Synechocystis salina* (NZ_JADEVV010000092.1). Accordingly, *Synechocystis* sp. strain Mab 20 was identified as *Synechocystis sp*. *algini* MNE. The obtained sequence has been deposited under the accession number ON864447 in the GenBank database. Also, the BLAST result revealed that our strain has the similarity with other deposited cyanobacterial strains on the Gene bank as *Crocosphaera watsonii* (NZ_AESD01000223.1, 92.72% similarity), Cyanobacterium endosymbiont of *Rhopalodia gibberula* ( NZ_AP018341.1, 92.36% similarity), *Crocosphaera chwakensis* (NZ_AAXW01000006.1, 92.37% similarity), *Crocosphaera subtropica* (NC_010546.1, 91.86% similarity), *Aphanothece sacrum* (NZ_BDQK01000013.1, 91.23% similarity), and *Microcystis flos-aquae* (NZ_JACJSW010000243.1, 91.21% similarity).

### Purity determination of the extracted polysaccharide by phytochemical analysis

Phytochemical tests for polysaccharide purity examination showed that BPS was free from any phytochemical compounds, including flavonoids, terpenoids, tannins, alkaloids, phlorotannins, glycosides, steroids, saponins, and phenols (Table 1). While a significant amount of phenol and saponins were detected in EPS and this problem can be overcome by subjecting extracted polysaccharides to defatting and deproteinization steps mentioned with BPS. Thus, phytochemical analysis displayed a higher purity of BPS than EPS. The presence of any accompanying cyanobacterial metabolites can be prejudicial to extracted polysaccharides further applied in several industries [52, 53].

**Table 1:** represents the phytochemical tests, physicochemical, and biochemical properties for polysaccharide extracted from *S. algini* MNE ON864447

Physicochemical properties		Biochemical tests		Qualitative phytochemical tests for polymer purity	
pH	8.2 ± 0.1	Carbohydrate test	70.1 ± 0.51%^1^ 75.2 ± 0.3%^2^	Flavonoids test	−Ve^4^
Color	Pale-yellow	Total protein	0.90 ± 0.1%	Alkaloids test	−Ve
Moisture content	16.01 ± 0.3%	Sulfate content	0	Tannins test	−Ve
Gelling with K_2_Cr_2_O_7_, and ZnSO_4_	+ Ve^3^	Phenol content	0.7 ± 0.32%	Phlorotannins test	−Ve
		Lipids test	0	Terpenoids and steroids test	−Ve
Gelling with KMnO_4_, and CuSO_4_	−Ve	Ash analysis	13.2 ± .5%	Saponins test	−Ve

(1); carbohydrate yield from carbazole assay, (2); carbohydrate yield from phenol–sulfuric acid assay, (3); + Ve indicates the positive result and (4); −Ve indicates the negative result

### Physico-chemical properties and biochemical composition of extracted polysaccharide

**Fig. 3:**
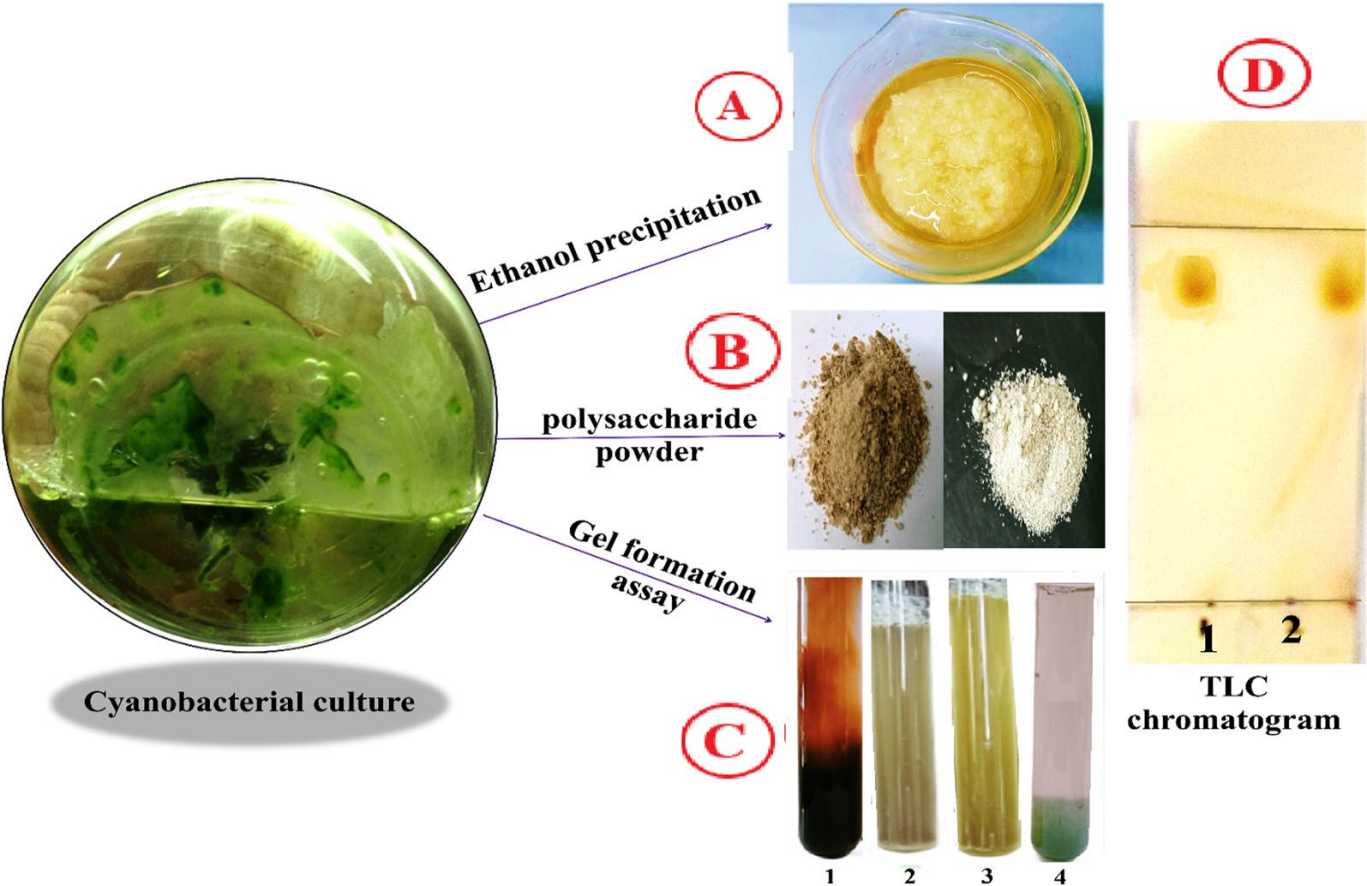
**A** Gelation of the cyanobacterial polymer extracted from* S. algini* MNE ON864447 after ethanol precipitation, **B** Color of purified cyanobacterial polymer powder against a white and black background, **C** Gel forming assay using, KMnO_4_ (1), ZnSO_4_ (2), K_2_Cr_2_O_7_ (3) and CuSO_4_ (4) solutions, **D** TLC chromatogram for hydrolyzed cyanobacterial polymer (1) and hydrolyzed alginate (2)

Table 1 displays the physicochemical properties of an extracted polysaccharide from *S. algini* MNE ON864447 including, color, pH, viscosity, gelling capacity, and moisture content. The color of the extracted polysaccharide after purification from ethanolic precipitation (Fig. 3A) showed a pale yellow color (Fig. 3B) and recorded a pH value of 8.2 ± 0.1 at a concentration of 1 g/100 mL. While the moisture content of the extracted polysaccharide equaled 16.01 ± 0.3%. Our study examined the effect of four divalent cation solutions; CuSO_4_, ZnSO_4_, K_2_Cr_2_O_7_, and KMnO_4_ on polysaccharide gelling capacity, and the result displayed that polysaccharide extracted from *S. algini* MNE ON864447 tends to form a gel with a solution of K_2_Cr_2_O_7_ and ZnSO_4_, while precipitation occurrs with CuSO_4_ and KMnO_4_ solutions (Fig. 3C). The gelling process can be generally induced by two factors: ionic strength and the formed gel termed as ionic-induced gels, and temperature and the formed gel, termed thermal-induced gels [54]. The gelling mechanism is known as the “egg-box” model [55]. The biochemical composition of polysaccharide extracted from *S. algini* MNE ON864447 is given in Table 1. Molisch’s and Iodine tests for extracted polysaccharides are positive, indicating the presence of carbohydrates. Also, the negative result obtained from the Seliwanoff test indicates the absence of ketoses and aldoses. The lack of Aldo-and keto-sugars in the EPS revealed the presence of other sugar units. Carbazole colorimetric assay was used to determine the presence of Uronic acids after polysaccharide hydrolysis using standard alginic acid. The result revealed that uronic acid constitutes 70.1 ± 0.51% of *S. algini* MNE ON864447 EPS. These results agreed with the outcome of the phenol–sulfuric acid method, as the extracted polysaccharide showed a carbohydrate yield of 75.2 ± 0.3%. Also, the other biochemical tests showed a significant presence of other compounds like ash, water, proteins, and phenols. Tentatively, depending on the overall physical and biochemical tests, we suggested that the extracted polysaccharide from *S. algini* MNE ON864447 is alginate, which is composed of mannuronic and guluronic acids, and it becomes necessary to confirm the chemical structure through other chemical analysis.

### TLC and HPLC analysis for *S. algini* MNE ON864447 polysaccharide

**Fig. 4:**
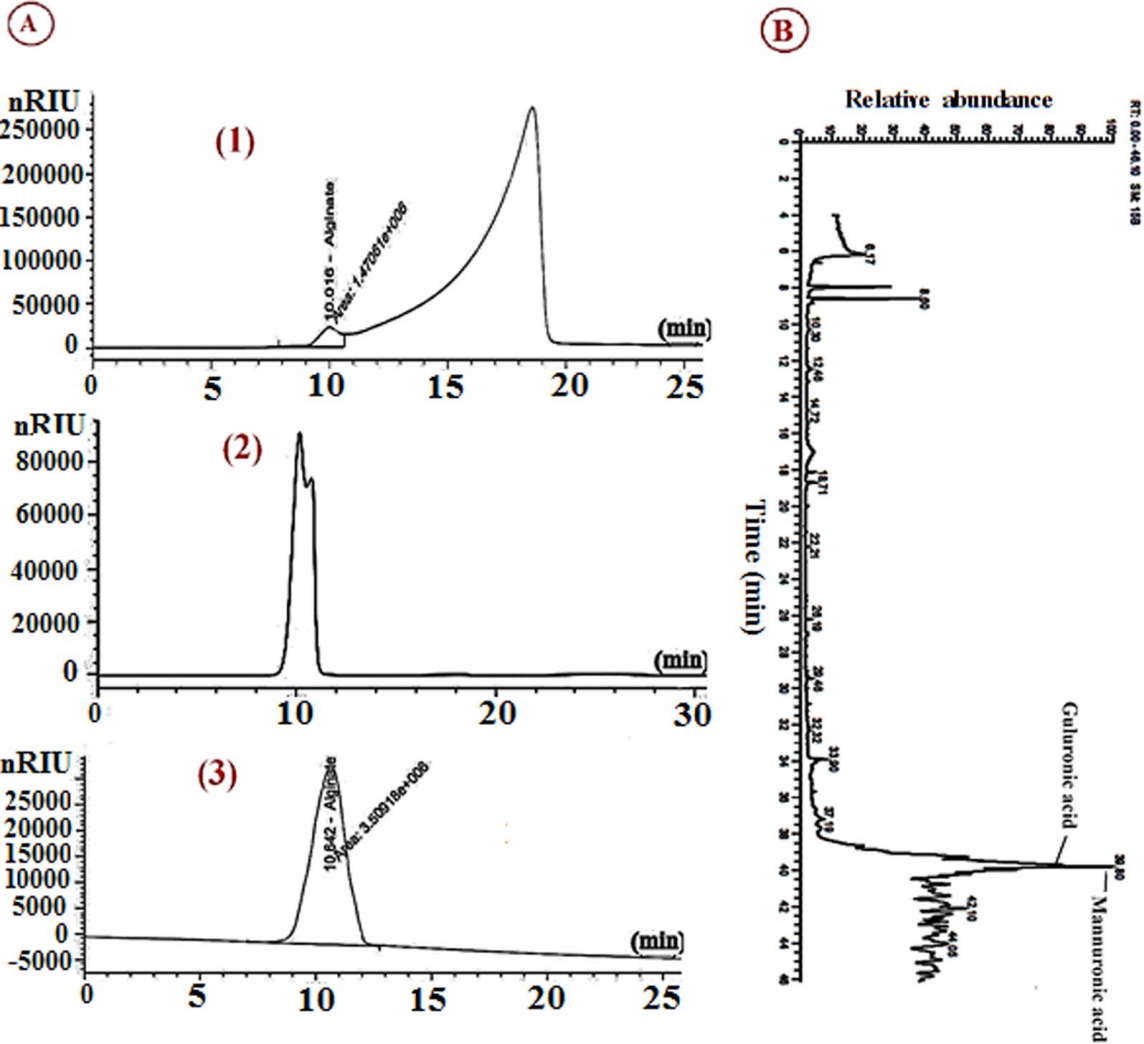
**A** High-performance liquid chromatography (HPLC) for cyanobacterial extracellular alginate from *S. algini* MNE ON864447 (1), cyanobacterial bounded alginate from* S. algini* MNE ON864447 (2), and standard alginate from *Macrocystis pyrifera* (3). **B** GC/MS chromatogram represents the monosaccharide constitutes of cyanobacterial alginate

TLC chromatogram showed (Fig. 3D) that hydrolyzed solutions of *S. algini* MNE ON864447 polysaccharide and standard alginate have the same retention factor (Rf); 0.82. Based on physical and chemical tests, we assume that *S. algini* MNE ON864447 polymer is alginate. HPLC was used to quantify and identify this polymer compared to standard alginate extracted from* Macrocystis pyrifera* through separate injection on an ion-exchange column, Aminex HPX-42A column, which is packed with a polymer-based matrix; polystyrene divinyl benzene. Aminex carbohydrate analysis columns separate compounds using a combination of size exclusion and ligand exchange mechanisms. The obtained chromatograms for the standard alginate (Fig. 4A-3) and cyanobacterial BPS (Fig. 4A-2) showed a single peak with a retention of 10.6 min for both. While HPLC chromatogram for cyanobacterial EPS showed two peaks; a small peak with a 1.47 area and a high peak is contaminated substance (Fig. 4A-1). Using the alginate calipiration curve, the alginate constitutes 75 ± 2.1% of the extracted polysaccharide. The HPLC method is a prime technique for compound identification and was found to be highly precise, sensitive, inexpensive, and accurate for the determination of alginate.

### GC–MS analysis for *S. algini* MNE ON864447 polysaccharide

Purified polysaccharide extracted from *S. algini* MNE ON864447 was subjected to trifluoroacetic (TFA) acid hydrolysis assisted by microwave, followed by mercaptalation and silylation, before being introduced to GC–MS analysis. The GC–MS chromatogram displayed complete baseline separations of all TMS-derivatized monosaccharides **(**Fig. 4B**)**. All peaks obtained from total ion chromatogram (TIC) analysis were identified by comparison of their mass spectra with those of WILEY 09 and NIST14 mass spectral database. Our chromatogram presented more than 7 peaks, which were the main peaks that appeared at a retention time of 39.6 and 39.8 min, corresponding to guluronic acid and mannuronic acid with a relative concentration of 36.2% and 44.3%, respectively. All the small peaks that appear in the chromatogram with unidentified compounds were considered uronic acid derivatization byproducts. Releasing all monosaccharides from polysaccharides is not easy and successful work, especially for uronic acid-involving polysaccharides since glycosidic bonds between their sugar units are acid resistant. The most common method for liberating monosaccharides is acidic hydrolysis. Trifluoroacetic acid (TFA) and sulfuric acid are two of the most commonly used reagents for acidic hydrolysis. For soluble polysaccharides like separated polysaccharides and secreted polysaccharides, TFA is most frequently utilized as it is volatile and its removal is easy. Although polymers that are difficult to fully hydrolyze or that are intractable, such as plant cell walls, are frequently treated with sulfuric acid [56]. Moreover, sulfuric acid-based hydrolysis requires additional purification to remove excess and involatile sulfuric acid, which makes the hydrolysis process more difficult and time-consuming. Microwave-assisted hydrolysis of polymeric sugars displayed its efficacy in complete glycosidic cleavage and liberation of monosaccharides from polysaccharides [57]. The simultaneous occurrence of carboxyl and hydroxyl groups in one molecule (uronic acids-containing polysaccharides) in addition to coupling with an anomeric center, diverse lactones will be obtained after derivatization, and consequently, complex chromatograms will be generated. We operate three different procedures; for *S. algini* MNE ON864447 polysaccharide GC–MS analysis and this method is the most successful procedure. The sugars content of many cyanobacterial EPS includes the pentoses ribose, arabinose, and xylose; the hexoses fructose, galactose, glucose, and mannose; the acidic hexoses galacturonic acids and guluronic; the deoxyhexoses rhamnose and fucose; the amino sugars N-acetyl galactosamine, N-acetyl glucosamine, galactosamine, and glucosamine [15]. The most prevalent monosaccharide in cyanobacterial EPS is often glucose. Nonetheless, certain cyanobacterial polymers have been reported to include larger concentrations of xylose, rhamnose, mannose, arabinose, uronic acids, and fucose than glucose [11]. For instance, the RPS of *Microcystis wesenbergii* was found to contain solely uronic acids [58]. It is an uncommon characteristic in microbial EPS, many cyanobacterial EPS also include two distinct uronic acids (from 2 up to 80% of the total EPS dry weight, often between 15 and 30%) [59].

### NMR analysis for *S. algini* MNE ON864447 polysaccharide

**Fig. 5:**
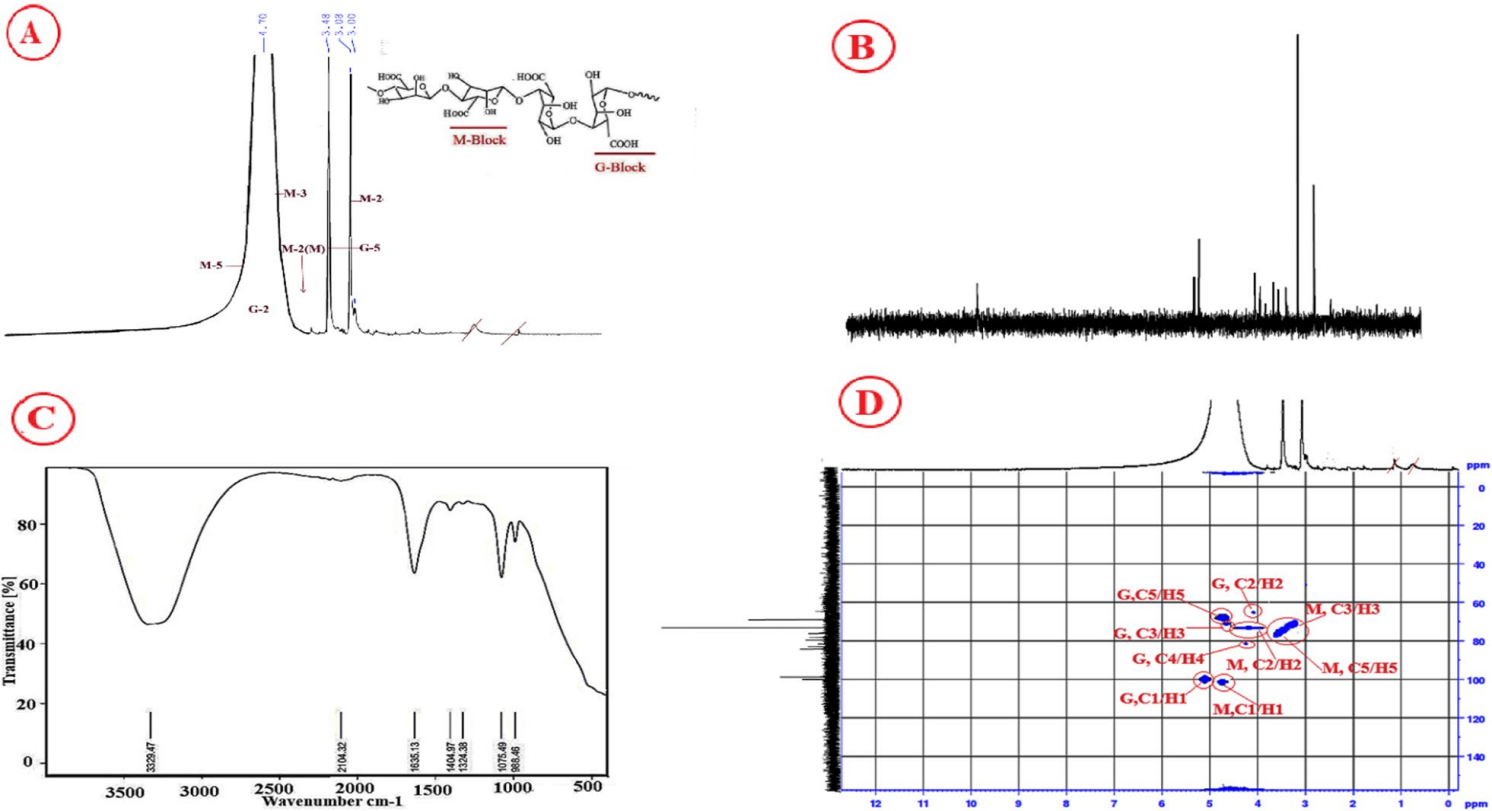
**A** H^1^-NMR analysis, **B** C.^13^ –NMR analysis, **C** FTIR spectra, and **D** HSQC analysis for cyanobacterial alginate produced by *S. algini* MNE ON864447

According to the ^1^H NMR spectrum resonances, sugar ring protons are present in the region between 3 and 4.4 ppm up-taking polysaccharides, and an anomeric proton is present in the region between 4.4 and 5.6 ppm [60]. The ^1^H spectra of isolated cyanobacterial alginate are shown in Fig. 5A; they revealed a large doublet-centered peak at 4.7 ppm, which was attributed to the anomeric proton resonances of guluronic acid residue (G) and manuronic acid residue (M), the two building blocks of alginate. These monomer arrangements can be in GG, MM, or GM blocks distributed in a non-regular order along the alginate. These findings matched those that had previously been reported for the sodium alginate fractions from *P. aeruginosa* and the algae *Macrocystis pyrifera*, respectively [61]. Alginate G5-H2 moieties of L-guluronic acid in homopolymeric blocks are attributed to the detected signal between 4.2 and 5.1 ppm [62]. A resonance below 3.2 ppm, a property of proteins and lipids, as well as acetyl and succinyl groups, was also detected in the spectrum fraction of cyanobacterial alginate [63, 64]. These broad bands at 4.7 ppm, 3.4 ppm, 3.08 ppm, and 5.1 ppm are confirmed to be strongly predictive of the presence of the G and M blocks of alginate in our documentary. The resonances were in good agreement with those published in the literature [63, 65]. The 13C NMR analysis (Fig. 5B) showed the chemical shifts of mannuronic and guluronic carbon atoms. The chemical shifts at 101, 71, 72.8, 78.5, 77, and 175 ppm was assigned for C1, C2, C3, C4, C5, and C6 of mannuronic acid residue. While the chemical shifts at 100, 65.9, 70.2, 80.55, 68.6, and 175 ppm were assigned for C1, C2, C3, C4, C5, and C6 of guluronic acid residue. The ^13^C-^1^ H HSQC NMR spectrum of partially degraded cyanobacterial alginate is seen in Fig. 5D. In heteropolymeric blocks, the predominant ^13^C signals were attributed to mannuronic and guluronic acids in the sequences GMG and MGM. In the anomeric region, connectivities between two ^13^C–^1^H systems were observed. The C-1/H-1 of the β-D-mannuronic acid residues in the triad GMG was assigned a correlation of 102.13/4.70 ppm, while the C-1/H-1 of the α-L-guluronic acid residue in the triad MGM was given a correlation of 100.51/5.06 ppm. Except for C-4/H-4 in mannuronic acid residues, correlations for the other ^13^C/^1^H systems were found and are displayed in Fig. 5D.

### FT-IR spectroscopic analysis for *S. algini* MNE ON864447 polysaccharide

The FT-IR spectrum of *S. algini* MNE ON864447 polysaccharide is presented in Fig. 5C. In the 3600–1600 cm^1^ region, three bands appear: a broad band centered at 3392.47 cm^1^ assigned to hydrogen-bonded O–H stretching vibrations, the weak signal at 2104.32 cm^1^ due to C–H stretching vibrations, and the asymmetric stretching of carboxylate O–C–O vibration at 1635.13 cm^1^. The band at 1404.97 cm^1^ may be due to C–OH deformation vibration with a contribution of O–C–O symmetric stretching vibration of the carboxylate group. (18,19) The weak bands at 1324.38, 1125.34 and 1075.49 cm^1^ may be assigned to C–C–H and O–C–H deformation, C–O stretching, and C–O and C–C stretching vibrations of pyranose rings, respectively. The fingerprint, or anomeric, region (950–750 cm^1^) is the most discussed in carbohydrates [66]. The spectrum shows a band at 988.46 cm^1^, which was assigned to the C–O stretching vibration of uronic acid residues, and one at 888.3 cm^1^ assigned to the C1–H deformation vibration of b-mannuronic acid residues. The band at 820.0 cm^1^ seems to be characteristic of mannuronic acid residues. The results of FTIR are remarkably in agreement with previous works [67–69]. The special characteristics of having dissimilar units of uronic acids made the polymer one of the functional gel-forming exopolysaccharides. The current frequency shift in the carbohydrate region strongly indicates the presence of mannuronic and guluronic acid residues.

### The M/G ratio of alginate extracted from *S. algini* MNE ON864447

The M/G ratio of the alginate from *S. algini* MNE ON864447 was found to be 1.02 ± 0.7. Also, the proportions of G-block, M-block, and MG-block constitute 2.2 ± 1.8, 2.9 ± 0.53, and 69.3 ± 0.5% of cyanobacterial alginate, respectively. Numerous researchers have noted that the M/G ratio and the block structure of alginate may significantly affect the substance's physical characteristics and the rheological characteristics of the associated gels [70]. Alginate's ability to produce gels is influenced by its M/G ratio, with high G-alginate producing stiff, brittle gels and high M-alginate producing soft, elastic gels [71].

### Statistical optimization for alginate production from *S. algini* MNE ON864447

#### Plackett–Burman design to identify significant factors affecting alginate production

**Fig. 6:**
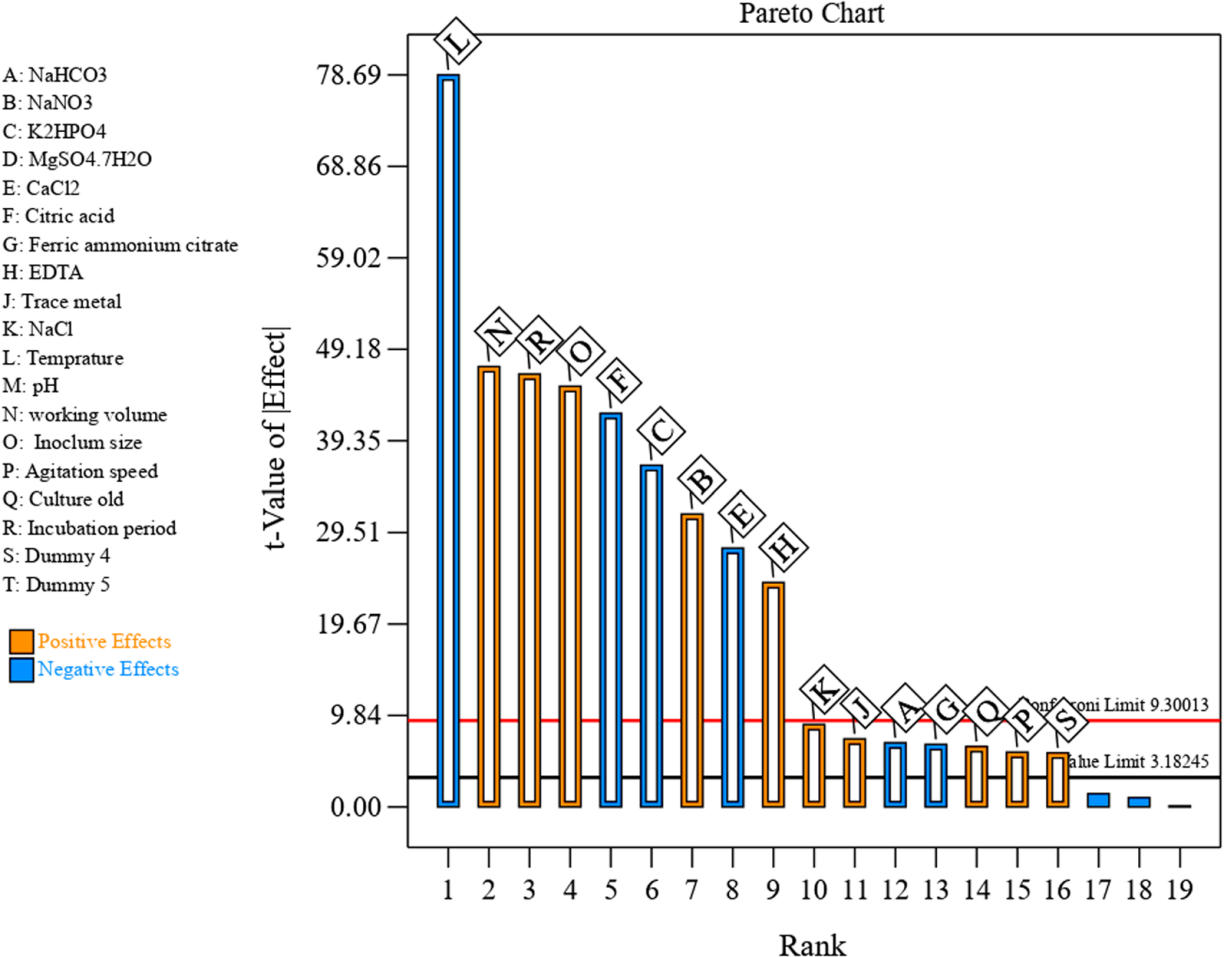
The Pareto chart evaluates the effects of independent variables on alginate production from *S. algini MNE ON864447* (the orange color represents the positive effect and the blue color represents the negative effect)

Seventeen independent factors including A (Sodium bicarbonate), B (sodium nitrate), C (dipotassium hydrogen phosphate), D (Magnesium sulfate hepta hydrate), E (Calcium chloride), F (Citric acid), G (Ferric ammonium citrate), H (EDTA), J (Trace metal), K (Sodium chloride), L (Temperature), M (pH), N (working volume), O (Inoculum size), P (Agitation speed), Q (Culture old), R (Incubation period) and two dummy factors were screened based on their impacts on the production of alginate from *S.* algini MNE ON864447 using 20 trials of Plackett–Burman experimental design (Additional file 1: Table S2). The results of the Plackett–Burman design under submerged fermentation (SF) conditions showed that, the minimum amount of alginate (3.099 mg/ml) was reached in run no. 12, while the maximum amount of alginate (12.52 mg/ml) was achieved in run no. 18. The statistical analysis of the Plackett–Burman design results determines the relationship between alginate production and the independent variables (Table 2). The data were interpreted based on the signs of the coefficients and primary effects (positive or negative impacts on alginate production). Based on the calculated major effects of the variables (Table 2), nine of the seventeen variables positively affect alginate production: working volume, incubation period, inoculum size, sodium nitrate, EDTA, NaCl, trace metal, culture age, and agitation speed (Fig. 6). The other eight variables (temperature, citric acid, K_2_HPO_4_, CaCl_2_, NaHCO_3_, Ferric ammonium citrate, MgSO_4_, pH) negatively affect alginate synthesis (Fig. 6). When the determination coefficient (R^2^) value is close to 1, the design utilized for predicting the response is better and stronger. In this investigation, the R^2^ value is 0.9998, suggesting that the model is a fit. Furthermore, the corrected R^2^ of 0.9990 is exceptionally high, indicating that the model is very significant. A higher predicted R^2^ (0.9928) indicates that this model is effective for predicting the amount of alginate production in the given range of variables.

**Table 2:** Plackett–Burman design statistical analysis for alginate production from *S. algini* MNE ON864447

Factor	Coefficient Estimate	Main effect	% contribution	F-value	P-value
Intercept	7.44			1153.53	< 0.0001
A-NaHCO_3_	−0.1276	−0.25	0.25	47.62	0.0062
B-NaNO_3_	0.5822	1.16	5.37	991.71	< 0.0001
C-K_2_HPO_4_	−0.6785	−1.35	7.29	1347.10	< 0.0001
E-CaCl_2_	−0.5145	−1.02	4.19	774.56	0.0001
F-Citric acid	−0.7817	−1.56	9.68	1788.06	< 0.0001
G-Ferric ammonium citrate	−0.1242	−0.24	0.244	45.17	0.0067
H-EDTA	0.4461	0. 89	3.15	582.27	0.0002
J-Trace metal	0.1352	0.27	0.28	53.46	0.0053
K-NaCl	0.1639	0.32	0.42	78.61	0.0030
L-Temperature	−1.45	−2.9	33.5	6192.35	< 0.0001
N-working volume	0.8747	1.74	12.12	2238.78	< 0.0001
O- Inoculum size	0.8355	1.67	11.06	2042.74	< 0.0001
P-Agitation speed	0.1088	0.217	0.18	34.61	0.0098
Q-Culture old	0.1201	0.24	0.22	42.21	0.0074
R-Incubation period	0.8599	1.719	11.72	2163.49	< 0.0001
S-Dummy 4	0.1075	0.214	0.18	33.79	0.0101
R^2^	0.9998	Std. Dev	0.0827		
Adjusted R^2^	0.9990	Mean	7.44		
Predicted R^2^	0.9928	C.V. %	1.11		
Adeq Precision	124.1373	PRESS	8.65

P and F-values were calculated to determine the importance of the model and the variables (Table 2). The ANOVA of the Plackett–Burman design indicated that the model is highly significant, as evident from a very low probability value (P = 0.0005) and the F-value of 2164.81. Also, the process factors with P-values less than or equal to 0.05 (confidence levels greater than or equal to 95 percent) were considered to have significant impacts. In the current investigation, the results revealed that fifteen variables are significant (P < 0.05) for alginate production. The results showed that, temperature (P < 0.0001 and F-value 12,346.02) was the most significant independent factor affecting the production of alginate by *S. algini* MNE ON864447; followed by working volume (F-value 4463.57 and P-value 0.0002), and incubation period (F-value 4313.47 and P-value 0.0002). The data displayed that; MgSO_4_.7H_2_O (D) and pH (M) are two non-significant independent factors (P˃0.05). The adequate precision value measures the signal-to-noise ratio. A value higher than 4 is desirable and indicates the good fit of the model. The adequate precision value of the present model is 170.34 and this value is adequate, so the model can be used to navigate the design space. The model showed PRESS, mean, standard deviation, and C.V. % (coefficient of variation) values of 68.5, 7.44, 0.058, and 78.7; respectively (Table 2). The regression coefficients were calculated (Table 2) and the data was fitted to the first-order polynomial equation (Eq. 1) to describe the relationship between the independent factors and the alginate production (Y) by *S. algini* MNE ON864447. The equation of the model fitted with a regression analysis was obtained in terms of the coded independent factors:

1 $${\text{Y}}\, = \, + \,{7}.{4}\, - \,0.{\text{127A}}\, + \,0.{\text{58B}}\, - \,0.{\text{678C}}\, - \,0.0{\text{26D}}\, - \,0.{\text{514E}}\, - \,0.{\text{78F}}\, - \,0.{\text{124G}}\, + \,0.{\text{45H}}\, + \,0.{\text{135J}}\, + \,0.{\text{16K}}\, - \,{1}.{\text{45L}}\, + \,0.{\text{87N}}\, + \,0.{\text{84O}}\, + \,0.{1}0{\text{9P}}\, + \,0.{12}\, + \,0.{\text{86R}}\, + \,0.{\text{11S}}$$

### Model adequacy checking

**Fig. 7:**
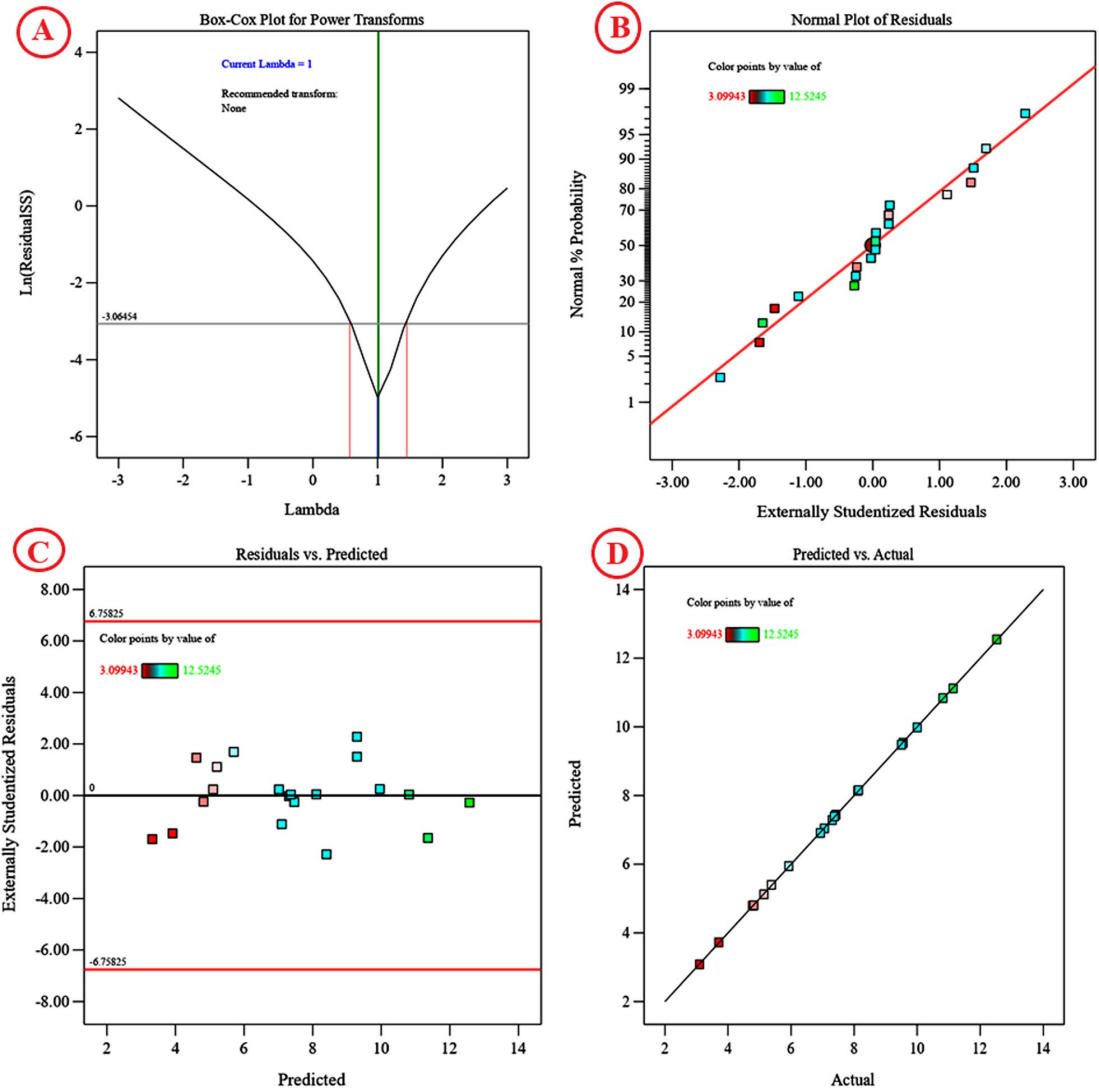
Plackett–Burman diagnostics for cyanobacterial alginate production, **A** Box–Cox plot of model transformation, **B** Normal probability plot of residuals, **C** plot of internally studentized residuals versus predicted values, and **D** plot of predicted versus actual values for cyanobacterial alginate producted from *S. algini* MNE ON864447

The normal probability plot (NPP) of the residuals is a critical graphical approach for visualizing the residuals’ distribution and determining the model’s adequacy [72]. The residuals are the difference between the theoretical model’s predicted response values and the experimental response values. A little residual value suggests that the model prediction is highly accurate and that the model fits the experimental results well. Figure 7B plots the NPP of the studentized residuals against the model's predicted response values. The residual points in this study are normally distributed; they are located next to the diagonal straight line, indicating the model's validity with the experimental results of the alginate production. Deviations from this straight line indicate that the residuals are not normally distributed. Figure 7D depicts a plot of predicted versus actual alginate production, with points near the fitted line, indicating a significant correlation between the model's predicted values and the experimental results of the alginate production, which confirms the accuracy of the model. The Box–Cox graph of the model transformation of the alginate production is shown in Fig. 7A. The green line represents the best lambda value (Lambda (λ) = 1) and the blue line represents the current transformation optimal value are stratify, indicating that the model is well fitted to the obtained experimental results. Therefore, the model is in the perfect zone, as the blue and green lines are stratified and in between the two vertical red lines (minimum and maximum values of confidence intervals between 0.45 and 1.45, respectively), indicating that the model fits the experimental data well and no data transformation is needed. A plot of the predicted alginate production vs. the studentized residuals is shown in Fig. 7C. The residuals were distributed uniformly and randomly above and below the zero line, with no distinct pattern, demonstrating that the residuals have a constant variance and validating the model’s accuracy.

### Face central composite design (FCCD) for optimization of alginate production

The face central composite design of response surface methodology (RSM) is an efficient experimental methodology for optimizing production conditions. The main advantage of RSM is that it involves a few experimental runs for different variables to obtain enough information for statistically acceptable findings. It is faster, saves time and effort, and prevents misunderstanding that happens with the conventional method. The RSM regression analysis facilitates finding significant variables, evaluating their correlations, and predicting the response [73]. FCCD determined the optimal levels of three variables: working volume (X1), incubation period (X2), and inoculum size (X3), as well as their mutual effects on alginate production. Variables with positive effects on alginate production have been used at high level, while the variables that have a negative effect are kept at low a level for further optimization by FCCD. The design matrix for 20 experimental runs is shown in Table 3, and the central point was repeated six times (2, 7, 9, 10, 12, and 13). Table 3 displays various combinations of the three independent variables and alginate production (predicted and observed values). The analysis indicates that there was a significant variation in alginate production based on the levels of the three independent variables. The central run no. 15, with a parameter of 500 ml working volumes, 20 days incubation periods, and 4% inoculum size, yielded the maximum alginate amount, with a value of 3.5743 g/L. The minimum alginate production (0.7739 g/L) was obtained in run number 17, which had a working volume of 600 ml, an incubation period of 20 days, and an inoculum size of 8%.

**Table 3:** Face central composite design for cyanobacterial alginate production from* S. algini* MNE ON864447 as influenced by working volume (X1), the incubation period (X2), and inoculum size (X3)

Std	Run	Factors			Total polysaccharide yield (g/L)		Residuals
		X1	X2	X3			
					Actual	Predicted	
14	1	0	0	1.68179	1.88983	1.95	−0.0632
17	2	0	0	0	1.395	1.45	−0.0564
13	3	0	0	−1.68179	3.23105	3.14	0.0869
4	4	1	1	−1	1.90191	1.95	−0.0501
12	5	0	1.68179	0	0.97914	0.9839	−0.0048
9	6	−1.68179	0	0	2.1476	2.15	−0.0009
19	7	0	0	0	1.49155	1.45	0.0402
5	8	−1	−1	1	1.96726	1.93	0.0334
20	9	0	0	0	1.49672	1.45	0.0454
18	10	0	0	0	1.42431	1.45	−0.0271
2	11	1	−1	−1	2.14052	2.18	−0.0405
16	12	0	0	0	1.45672	1.45	0.0054
15	13	0	0	0	1.43983	1.45	−0.0115
11	14	0	−1.68179	0	1.64845	1.62	0.0284
1	15	−1	−1	−1	3.57431	3.63	−0.0533
8	16	1	1	1	2.26586	2.23	0.0366
6	17	1	−1	1	0.77397	0.7749	−0.0009
3	18	−1	1	−1	1.40105	1.42	−0.0158
10	19	1.68179	0	0	1.64845	1.62	0.0245
7	20	−1	1	1	1.43019	1.41	0.0238

Level	Working volume (mL)	The incubation period (days)	Inoculum size (%)
−1.68	300	15	2
−1	400	20	4
0	500	25	6
1	600	30	8
1.68	700	35	10

### Statistical analysis of the data

Using design expert software, the FCCD experimental data were analyzed using multiple regression analysis. The model determination coefficient (R2) of 0.9964 indicates that the model could explain 99.64% of the variation in alginate production. Data were interpreted based on significance (P < 0.05). Statistical analysis reveals that the model is highly significant, according to Fisher's F-value (303.79) and a very low P-value (0.0001 < 0.05). The significance values (Table 4) demonstrate that the linear coefficients of working volume (X1), incubation period (X2), inoculum size (X3), and their quadratic effects are significant. Furthermore, the different interactions between the three variables investigated (X1X2; X1 X3, and X2 X3) are significant (*P*-values of < 0.0001, 0.0044, < 0.0001; respectively), demonstrating that they significantly contributed to the increase in alginate production. The quadratic model summary statistics had the greatest adjusted (0.9931) and predicted *R*^2^ (0.9777), as well as the lowest standard deviation (0.055). The equation of the second-order polynomial model (Eq. 2) has been proposed to analyze the association between different variables and determine the maximum alginate production corresponding to the optimal level of working volume, incubation period, and inoculum size. The maximum alginate production is predictable based on the optimal levels of the independent variables (X1, X2, and X3):

2 $${\text{Y}}\, = \,{1}.{45136}\, - \,0.{155967}\,*\,{\text{X}}_{{1}} \, - \,0.{189112}\,*\,{\text{X}}_{{2}} \, - \,0.{354121}\,*\,{\text{X}}_{{3}} \, + \,0.{495451}\,*\,{\text{X}}_{{1}} {\text{X}}_{{2}} \, + \,0.0{719136}\,*\,{\text{X}}_{{1}} {\text{X}}_{{3}} \, + \,0.{42}0{836}\,*\,{\text{X}}_{{2}} {\text{X}}_{{3}} \, + \,0.{153743}\,*\,{\text{X}}_{{1}}^{{2}} \, + \, - 0.0{528137}\,*\,{\text{X}}_{{2}}^{{2}} \, + \,0.{387942}\,*\,{\text{X}}_{{3}}^{{2}}$$

**Table 4:** Statistical analysis for FCCD results of cyanobacterial alginate production from* S. algini* MNE ON864447

Source	Sum of squares	df	Mean Square	F-value	p-value	Coefficient estimate
Model	8.47	9	0.9410	303.79	< 0.0001	1.45
A-Working volume	0.3322	1	0.3322	107.25	< 0.0001	−0.1560
B-Incubation period	0.4884	1	0.4884	157.68	< 0.0001	−0.1891
C- Inoclum size	1.71	1	1.71	552.88	< 0.0001	−0.3541
AB	1.96	1	1.96	633.97	< 0.0001	0.4955
AC	0.0414	1	0.0414	13.36	0.0044	0.0719
BC	1.42	1	1.42	457.39	< 0.0001	0.4208
A^2^	0.3406	1	0.3406	109.97	< 0.0001	0.1537
B^2^	0.0402	1	0.0402	12.98	0.0048	−0.0528
C^2^	2.17	1	2.17	700.18	< 0.0001	0.3879
Residual	0.0310	10	0.0031			
Lack of Fit	0.0232	5	0.0046	3.00	0.1264	
Pure Error	0.0077	5	0.0015		Adeq Precision	72.4880
Std. Dev	0.0557		R^2^	0.9964		
Mean	1.79		Adjusted R^2^	0.9931		
C.V. %	3.12		Predicted R^2^	0.9777		

Y = 1.45136—0.155967 * X_1_—0.189112 * X_2_—0.354121 * X_3_ + 0.495451 * X_1_X_2_ + 0.0719136 * X_1_X_3_ + 0.420836 * X_2_X_3_ + 0.153743 * X_1_^2^ + -0.0528137 * X_2_^2^ + 0.387942 * X_3_^2^ (2).

Where Y is the value of predicted production of alginate, X_1_ is the coded value of working volume, incubation period (X_2_), and inoculum size (X_3_).

### Model accuracy checking

**Fig. 8:**
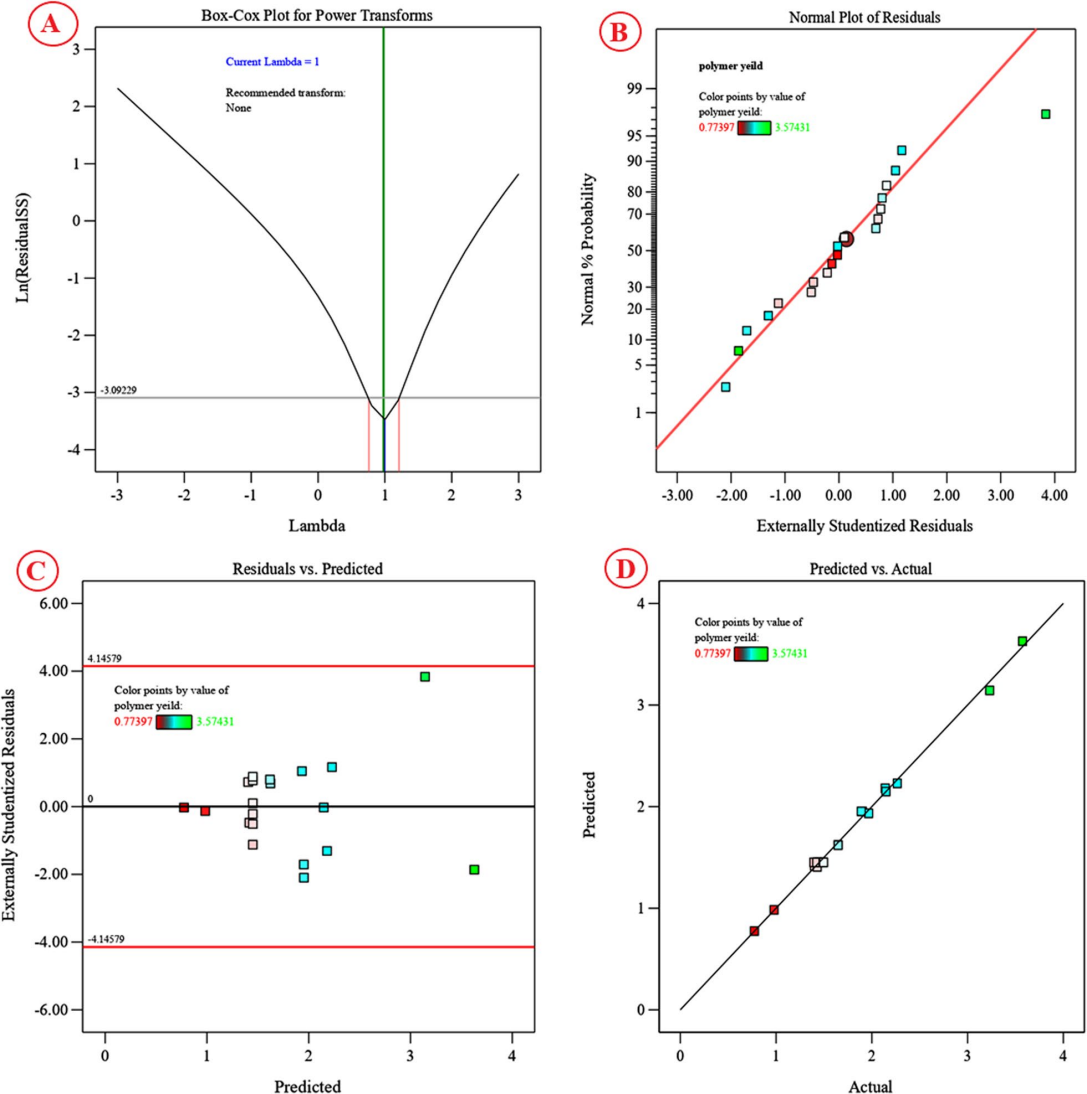
Face Central composite diagnostics for cyanobacterial alginate production from* S. algini* MNE ON864447, **A** Box–Cox plot of model transformation, **B** Normal probability plot of residuals, **C** plot of internally studentized residuals versus predicted values, and **D** plot of predicted versus actual values using Design Expert version 13 for Windows software

Some statistics were carried out for confirmation of the design’s accuracy. Figure 8A shows the Box–Cox graph, where the green line represents the best lambda value (Lambda = 1) and the blue line represents the current transformation (Lambda = 1). While the red lines represent the lowest and highest values of confidence intervals between 0.8 and 1.2. As Lambda’s best value lies between the minimum and maximum confidence intervals, no transformations of the data were recommended. The blue line lies between the two red lines; it implies that the model is well fitted to the experimental data obtained. Figure 8B displays the normal probability plot (NPP) of the residuals. The residuals have been drawn versus the normal scores, which are the cumulative probabilities. The resulting points are shown in the NPP of the residues close to the diagonal line in such a way that they are normally distributed, indicating a good model fit. Figure 8C shows a plot of the predicted alginate production against the residuals. The residuals scattered uniformly and randomly above and below the centre line of zero with no obvious pattern, indicating that the residuals have a constant variance, confirming the model’s adequacy. Figure 8D shows a plot of predicted versus actual alginate production, showing the points close to the fitted line, supporting a significant correlation between the predicted alginate production of the model and its actual results.

### Three-dimensional surface plots

**Fig. 9:**
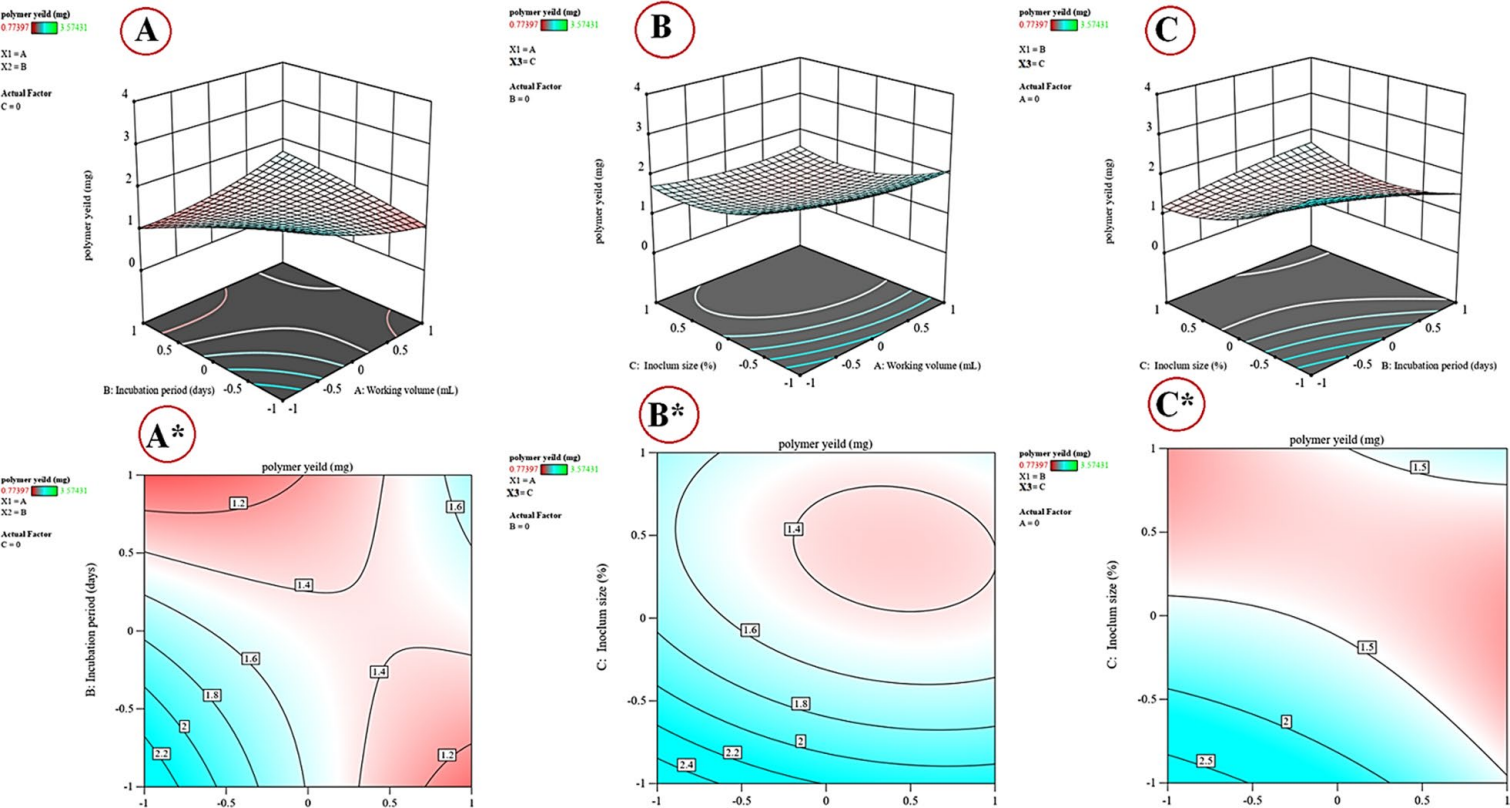
**A**–**C** 3D plots and contour (A*–C*) created using Design Expert version 13 for Windows software showing the effect of interaction between working volume (X_1_), incubation periods (X_2_), and inoculum size (X_3_) on the cyanobacterial alginate production from* S. algini* MNE ON864447

The three-dimensional surface plots were designed to identify the optimal concentrations and the interaction between the factors for maximal alginate production. Alginate production was plotted on the Z-axis against two factors, while the third factor was set at its zero level (Fig. 9A–C*). Figure 9A, A* illustrates the production of alginate as a response to working volume (X_1_), and incubation periods (X_2_) by keeping inoculum size (X_3_) at zero level. The results showed that higher levels of both working volume and incubation period were associated with lower alginate production. The alginate production increased gradually as both levels of the working volume (X_1_), and incubation periods (X_2_) decreased. By solving Eq. (2), the highest production of 3.57 g/L alginate could be achieved in 400 ml working volume at the optimal predicted 20 days of incubation period and 4% inoculum size. Working volume significantly affected the volumetric dissolved oxygen coefficient (KLa); the smaller the working volume, the higher the dissolved oxygen coefficient, which accelerated bacterial growth while simultaneously increasing substrate consumption rates [74, 75]. Conversely, less dissolved oxygen in the medium resulted in delayed microbial development, prolonged the fermentation cycle, and increased the time needed to produce alginate. Too little working volume resulted in a deficiency of nutrients necessary for subsequent microbial growth. Aeration and mixing are critical parameters for optimal polysaccharide synthesis. The previous investigation discussed the importance of agitation and mixing as the free alginate produced in *P. aeruginosa* culture limits the diffusion of O_2_ and consequently restricts bacterial growth and alginate production. The important role of O_2_ concentration constituted in controlling several alginate biosynthetic enzymes and genes. Furthermore, epimerase enzymes, specifically the gene algE2, which is responsible for introducing guluronic acid blocks into alginate, are oxygen-dependently up-regulated [76, 77]. It is important to point out that dissolved oxygen tension (DOT) may be regulated, either by adjusting the agitation rate of the culture or by varying the levels of nitrogen or oxygen present in the gas inflowing through mass flow controls [76, 78]. Also, previous studies reported that the dissolved oxygen tension (DOT) which has been manipulated by adjusting the agitation rate of the bioreactor, rendering it impossible to distinguish between the influence of oxygen in the bulk liquid and the agitation speed on alginate synthesis. Data obtained under non-nitrogen-fixing [79] and nitrogen-fixing conditions [77] show that dissolved oxygen tension (DOT), and culture stirring speed have a strong influence on alginate synthesis and polymer molecular mass. Pena et al. [76] demonstrated that in cultures run at 300 rpm, bacteria synthesized more alginate (4.5 g/L) under high DOT (5% air saturation) than under low (0.5%) oxygen tension (1.0 g/L).

Sabra et al. [77] observed that in phosphate-limited continuous culture, the specific rates of oxygen consumption and alginate formation of *A. vinelandii* increased as a function of culture DOT, yielding a specific alginate production rate (q_alg_) of 0.2 g/g.h at a dilution rate of 0.22 h-1 at 5% DOT. According to Sabra et al. [77], under nitrogen-fixing circumstances, the bacteria construct an alginate capsule, the composition of which varies according to the external DOT, which may assist in protecting the nitrogenase system from oxygen damage. Trujillo-Roldán et al. [79] demonstrated that alginate polymerization proceeds by producing chains with very uniform molecular mass distributions and little dispersion throughout the culture, independent of culture time or strain (wild type or AlgL mutant). Furthermore, DOT has a considerable effect on the mean molecular mass (MMM) of alginate produced by the* Azotobacter* family. This study demonstrated that DOT significantly affects the polymerase enzyme. DOT probably influences the transcription of alg8, which encodes the polymerase enzyme, similarly as it does for algA, algC, and algD in *P. aeruginosa* [80]. According to Trujillo-Roldán et al. [79], alginate lyase is not necessary for the synthesis of alginate; however, when it is present (as in the wild type), its function is limited to a post-polymerization step, with its activity maximized in the pre-stationary phase of development. A decrease in the MMM of the alginate is indicative of alginate-lyase activity.

Figure 9B, B* illustrates alginate production as a response to working volume (X1), and inoculum size (X3) by keeping the incubation period (X2) at zero level. At the highest levels of both working volume (X1), and inoculum size (X3), minimum alginate production has been obtained and a gradual increase in the production of alginate has been observed at lower levels of both working volume, and inoculum size. By analyzing Fig. 8B and predicting Eq. (2), the alginate production of 3.57 g/L could be attained after 20 days incubation period at the optimum predicted levels of working volume (400 ml) and inoculum size (4%). [81] found that the density of the inoculum or seed culture had a significant impact on the duration of the lag phase, specific growth rate, biomass, end product quality, and cost of production. As a result, they provided a clear definition of the fermentation process' production and economics. We hypothesized that high initial cell densities in the production medium might cause rapid oxygen depletion and the loss of other nutrients. As a result, cell aggregates and low growth rates may occur from limitations in dissolved oxygen and certain nutrients. Figure 9C, C* illustrates alginate production as a response to the incubation period (X2), and inoculum size (X3) by keeping the working volume (X1) at zero level. It has been shown that the larger production of alginate has been achieved at a lower level of working volume (X2) and inoculum size (X3). At a higher working volume (X2) level, medium alginate production has been obtained and increased as the working volume (X2) level decreased. While the maximum alginate production has been observed at the lowest inoculum size (X3) and production gradually decreases as the minimum alginate production is detected at a higher inoculum size. By analyzing Fig. 8C and predicting Eq. (2), the maximal cyanobacterial alginate production of 3.57 g/L could be achieved with 4% inoculum and the optimal predicted levels of working volume (400 mL) and incubation period (20 days). The decline phase of the cyanobacteria occurred at 25 days, and a significant decrease in biomass during this period might be due to depletion of the nutrients and accumulation of toxic metabolites in the culture medium, which led to a gradual increase in the rate of microbial death and a decrease in viable bacteria. The bacterial cells lysed at the end of the decline phase, and AlgL, an alginate lyase enzyme, degraded the alginate complex [82].

### Antibacterial activity of alginate extracted from S. algini MNE ON864447

**Fig. 10:**
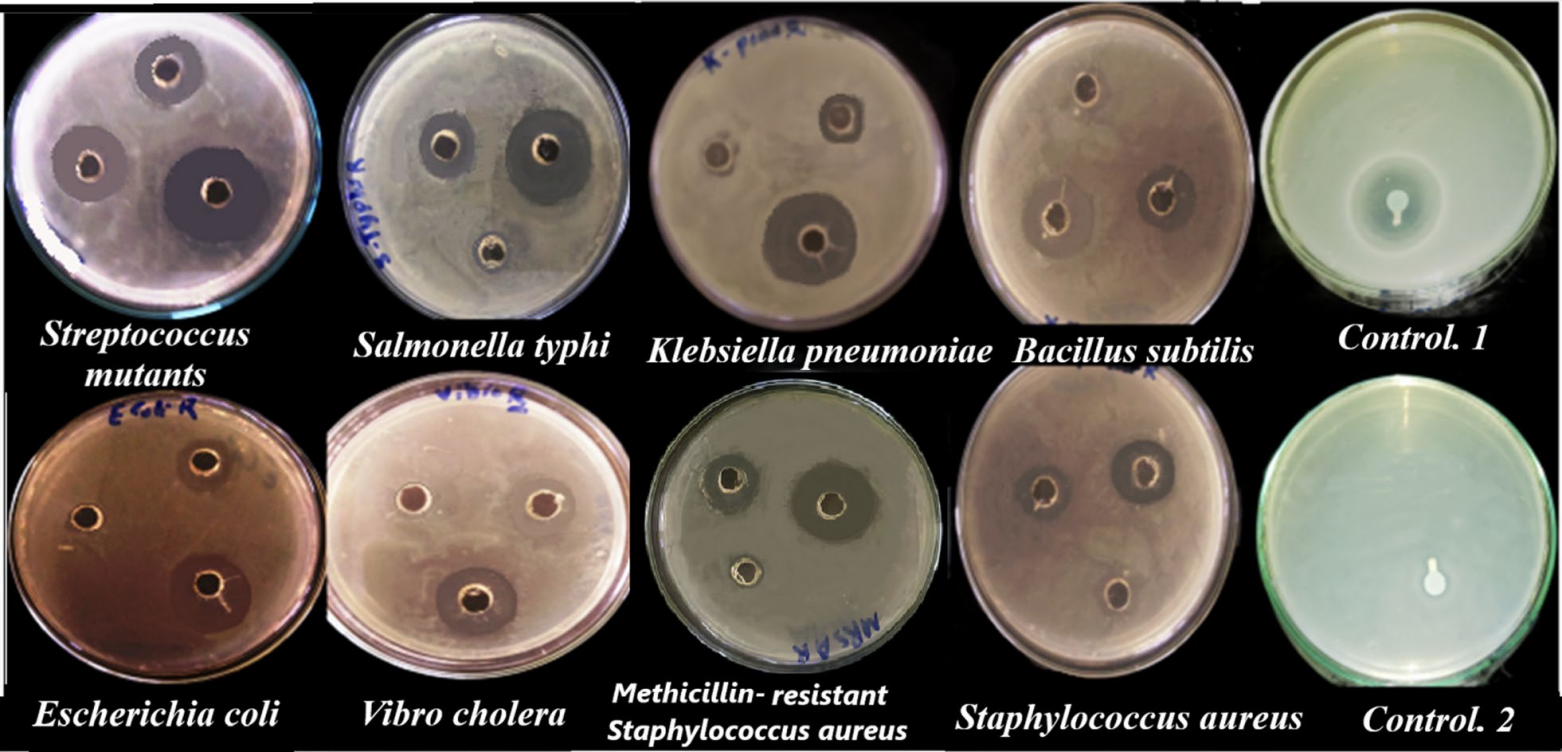
Antibacterial activity using agar-diffusion assay of alginate extracted from *S. algini* MNE ON864447 against different Gram-positive and Gram-negative bacteria

Alginate extracted from *S. algini* MNE ON864447 displayed antibacterial activity against both Gram-positive and Gram-negative bacteria and produced concentration-dependent inhibition zones (Fig. 10). The mean diameter of the inhibition zone (IZD) of cyanobacterial alginate evaluated against the test organisms ranged between 10.53 ± 0.03 mm against *Vibro cholera (NCTC 8021)* at a concentration of 2.5 mg/mL and 34 ± 0.1 mm against *Streptococcus mutants (NCTC10449)* at a concentration of 10 mg/mL. Table 5 showed that the growth of Gram-positive bacteria, including *Staphylococcus aureus (ATCC25923),* Methicillin resistance* Staphylococcus aureus* (MRSA)*, Streptococcus mutants (NCTC10449) and Bacillus subtilis (NCTC 10400),* was inhibited by cyanobacterial alginate at concentrations of 2.5–10 mg/mL. *Staphylococcus aureus* (ATCC25923) was sensitive to cyanobacterial alginate and showed inhibition zone diameters of 24.3 ± 0.2, 21.2 ± 1, 12.1 ± 0.13 mm at a concentration of 10, 5, and 2.5 mg/mL, respectively. *Streptococcus mutants* (NCTC10449) showed susceptibility to cyanobacterial alginate with inhibition zone diameters of 34 ± 0.1, 26.5 ± 0.05, 21.5 ± 0.15 mm, and 23 ± 0.05, 17.5 ± 0.15, 10.7 ± 0.15 mm with* Bacillus subtilis* (NCTC 10400) at concentrations of 10, 5, and 2.5 mg/mL, respectively. The inhibitory activity of cyanobacterial alginate was also observed against Gram-negative *Klebsiella pneumoniae* (IZD*,* 28.5 ± 0.05, 14.9 ± 0.01, 11 ± 0.1 mm at cyanobacterial alginate concentrations of 10, 5, and 2.5 mg/mL, respectively), *Vibro cholera* (NCTC 8021) (IZD*,* 22.5 ± 0.05, 16 ± 0.1, 10.53 ± 0.03 mm at cyanobacterial alginate concentrations of 10, 5, and 2.5 mg/mL, respectively),* Salmonella typhi* (ATCC 19430) (IZD, 31.5 ± 0.15, 18.5 ± 0.15, 11.5 ± 0.1 mm at cyanobacterial alginate concentrations of 10, 5, and 2.5 mg/mL, respectively), and* Escherichia coli* (ATCC 25922) (IZD, 28.5 ± 0.15, 21.5 ± 0.15, 11 ± 0.08 at cyanobacterial alginate concentrations of 10, 5, and 2.5 mg/mL, respectively). These results are compared to standard streptomycin, which showed activity against Gram-positive bacteria; *Salmonella typhi* (ATCC 19430) with 34 mm inhibition zone diameter (Fig. 10 control 1) and no activity was detected against MRSA (Fig. 10 control 2). We suggested that the negative charge of the alginate molecule plays a key role in its antimicrobial activity, as reported in the literature. Asadpoor and his coworker reported that alginate oligosaccharides (AOS) have a potent inhibitory effect on the growth of *Streptococcus agalactiae* (GBS), even at low concentrations (AOS 1%) [20]. The anionic nature of AOS may be responsible for its antibacterial activity. AOS significantly inhibited *S. aureus* wood 46 growth, suggesting that its action may be strain-dependent. Interestingly, Hu et al. [83] reported that a depolymerized product of alginate (a mannuronic acid derivative) displayed high inhibitory activity against *S. aureus*. The variation in AOS's anti-pathogenic actions may be due to a match or mismatch between its negatively charged structural characteristics and the target structures of various bacterial strains. Furthermore, anti-biofilm properties of AOS against Gram-negative bacteria like *P. aeruginosa* were reported [84, 85]. Moreover, Asadpoor and his colleague showed that AOS significantly decreased the biofilm formation of both GBS (4, 8, and 16% AOS) and *S. aureus* wood 46 (8 and 16% AOS) [20]. Polysaccharide intercellular adhesin (PIA) is one of the most significant positively charged polymers in *Staphylococci* sp. biofilms. PIA plays a crucial role in intercellular adhesion and is implicated in at least the majority of infections linked to staphylococcal biofilms. At neutral or basic pH, the multivalent electrostatic interactions of the cationic PIA polymer with the negatively charged bacterial cell surface (such as negatively charged teichoic acids) affect the negatively charged bacterial cell surface. Alginates can obstruct this intramolecular interaction in EPS [86]. According to Asadpoor [20], alginates can interfere with intramolecular interactions in EPS (such as mucus) and competitively hinder the interpolymer cross-links, weakening the structures of biofilms.

**Table 5:** Inhibition zone diameter (mm) produced by alginate extracted from* S. algini* MNE ON864447 against Gram-negative and Gram-positive bacteria

Bacterial strains	Cyanobacterial alginate concentrations		
	10 mg/ml	5 mg/ml	2.5 mg/ml
*Klebsiella pneumoniae*	28.5 ± 0.15	14.9 ± .01	11 ± 0.1
*Vibro cholera (NCTC 8021)*	22.5 ± .05	16 ± 0.1	10.53 ± 0.03
*Escherichia coli (ATCC 25922)*	28.5 ± 0.15	21.5 ± 0.15	11 ± 0.08
*Bacillus subtilis (NCTC 10400)*	23 ± .05	17.5 ± 0.15	10.7 ± 0.15
*Salmonella typhi (ATCC 19430)*	31.5 ± 0.15	18.5 ± 0.15	11.5 ± 0.1
*Streptococcus mutants (NCTC10449)*	34 ± 0.1	26.5 ± .05	21.5 ± 0.15
*Staphylococcus aureus (ATCC25923)*	24.3 ± 0.2	21.2 ± 1	12.1 ± 0.13
*MRSA*	28.5 ± 0.15	21.5 ± 0.15	11.1 ± .02

## Conclusion

This is the first report about the production of alginate from a new cyanobacterium isolate; *S. algini* MNE ON864447 that can overcome the limitations of alginate production from brown algae, such as lower abundance of brown algae and a lack of uniform alginate quality. As well, our results revealed that *S. algini* MNE ON864447 produced an alginate polymer with a 1.02 ± 0.7 M/G ratio that was able to form cross-links with divalent cations. The color of the extracted alginate is a pale yellow color, and it recorded a pH value of 8.2 ± 0.1 at a concentration of 1 g/100 mL. Uronic acid constitutes 70.1 ± 0.51%, while the moisture content constitutes 16.01 ± 0.3% of cyanobacterial alginate. The defatting process and deproteinization with sevag solution get rid of associated contaminants in alginate polymer. Screening of seventeen nutritional and environmental variables on alginate production using the Plackett–Burman design revealed that the working volume, the incubation period, and the inoculum size are the most significant factors affecting alginate production from *S. algini* MNE ON864447. Face central composite design (FCCD) showed a three-fold increase in alginate production in an optimized medium compared to an unoptimized one. Cyanobacterial alginate showed antibacterial activity against both Gram-negative and Gram-positive bacteria at concentrations ranging from 2.5 to 10 mg/mL and the most potent activity was detected against *Streptococcus mutants* (NCTC10449) and *Salmonella typhi* (ATCC 19430). Cyanobacteria can be considered an alternative source of alginate that can be applied in biomedical, industrial, ecological, and technological sectors as drug carriers, matrices in the textile and paint industries, metal chelators in bioremediation processes, and so on.

### Supplementary Information

**Additional file 1: Table S1.** Morphological characteristics of cyanobacterial isolates and their exopolysaccharide yield. **Table S2**. Plackett–Burman experimental design under SF fermentation for cyanobacterial alginate production.

## Data Availability

Data will be made available on request.
