# Supplementary material for: Identification and statistical optimization of a novel alginate polymer extracted from newly isolated Synechocystis algini MNE ON864447 with antibacterial activity
Source: Microb Cell Fact. 2023 Nov 7;22:229. doi: 10.1186/s12934-023-02240-w (PMC10629183; doi:10.1186/s12934-023-02240-w)
Supplement: Supplementary file 1 — Additional file 1: Table S1. Morphological characteristics of cyanobacterial isolates and their exopolysaccharide yield. Table S2. Plackett–Burman experimental design under SF fermentation for cyanobacterial alginate production. [file 12934_2023_2240_MOESM1_ESM.pdf]

Table S1. Morphological characteristics of cyanobacterial isolates and their exopolysaccharide yield.

Isolate code	Source of isolation	Culture color	General shape	Vegetative cell	Heterocyst		Sheath	Genus name	Exo-polysaccharide yield	
					Shape	Site			BPS	RPS
**Mab 1**	Cultivated soil	Bright blue-green	Filamentous	Long cylindrical with a [illegible]	-	-	-	*Pesudoanabena* sp.	0.45 (43%)*	-
**Mab 2**	Moist soil from rice field	Brown	Filamentous	spherical	spherical	Inter. &Ter.	-	*Nostoc* sp.	0.72 (75%)*	-
**Mab 3**	Non-cultivated	Olive green	Filamentous	Disc-shape	-	-	Thin	*Oscillatoria* sp.	0.0352 (16%)*	-
**Mab 4**	Agriculture wastewater	Light blue-[illegible]	Unicellular	Spherical	-	-	-	*Microcystis* sp.	0.533 (65%)*	-
**Mab 5**	Cultivated soil	Pale green	Filamentous	Disc-shape	-	-	Very thin	*Oscillatoria* sp.	0.031 (15%)*	-
**Mab 6**	Cultivated [illegible]	Orange [illegible]	Unicellular	Tetrad	-	-	-	*Tetraedron* sp.		1.01 (93%)*
**Mab 7**	Tap water	Light blue-green	Unicellular	Spherical	-	-	-	*Microcystis* sp.	0.445 (63%)*	-

**Mab 8**	Agriculture wastewater	Olive green	Unicellular	Spherical	-	-	-	*Chlorella* sp.	-	0.32 (20%)*
**Mab 9**	Cultivated [illegible]	Brown	Filamentous	Spherical	Spherical	Inter. [illegible]	-	*Nostoc* sp.	0.52 [illegible]	-
**Mab 10**	Sea water	Blue-	Coiled	Disc-shape	-	-	-	*Spirulina* sp.	0.79	-
**Mab 11**	Non-	Blue-	Filamentous	Cylindrical	Spherical	Inter.	-	*Anabaena* sp.	0.33	-
**Mab 12**	Drainage	Pale	Filamentous	Elongated	-	-	-	*Limnothrix* sp.	0.41	-
**Mab 13**	Irrigation canal	Blue-green	Filamentous	Cylindrical			Fine	*Phormidium* sp.	0.25 (12.7%)*	-
**Mab 14**	Agriculture wastewater	Blue-green	Filamentous	Spherical	Spherical	Inter. &Ter.	-	*Anabaena* sp.	0.034 (11.2%)*	-
**Mab 15**	Moist soil	Dark green	Filamentous	Disc- shape	-	-	Thick	*Lyngbya* sp.	0.23 (14.33%)*	-
**Mab 16**	Tap water	Blue-	Unicellular	Spherical	-	-	-	*Synechocystis*	-	0.65 (92%)*
**Mab 17**	Cultivated soil	Bright blue-green	Filamentous	Long cylindrical with a rounded end	-	-	-	*Pesudoanabena* sp.	0.52 (40.3%)*	-

**Mab 18**	Sewage water	Dark green	Unicellular	Spherical	-	-	-	*Chlorella* sp.	-	0.32 (19.5%)*
**Mab 19**	Tap water	Blue-green	Unicellular	Spherical	-	-	-	*Microcystis* sp.	0.45 (54.5%)*	-
**Mab 20**	Nile river	Blue-green	Unicellular	Spherical			-	*Synechocystis* sp.		1.2 (93.2%)*
**Mab 21**	Moist soil	Blue-green	Filamentous	Short cylindrical	-	-	Fine	*Phormidium* sp	0.22 (11.5%)*	-
**Mab 22**	Irrigation channels	Blue-green	Unicellular	Spherical	-	-	Present	*Synechococcus* sp.		0.91 (88%)*
**Mab 23**	Sandy soil	Blue-	Filamentous	Disc- shape	-	-	Very	*Oscillatoria* sp.	0.35	-
**Mab 24**	Sea water	Blue-	Filamentous	Barrel	Ovoid	Inter.	-	*Nostoc* sp.	0.66	-
**Mab 25**	Cultivated	Dark	Filamentous	Disc- shape	-	-	Thick	*Lyngbya* sp.	0.33	-

(*) indicates the polysaccharide yield as a percentage of initial cyanobacterial biomass.

Table S2. Plackett–Burman experimental design under SF fermentation for cyanobacterial alginate production.

Std	Run	A	B	C	D	E	F	G	H	J	K	L	M	N	O	P	Q	R	S	T	Total polysaccharide yield (mg/ml)		Residuals
																					**Actual**	**Predicted**	
9	1	1	-1	-1	-1	-1	1	1	-1	1	1	-1	-1	1	1	1	1	-1	1	-1	9.55477	9.51	0.0464
13	2	1	-1	1	-1	1	-1	-1	-1	-1	1	1	-1	1	1	-1	-1	1	1	1	7.0592	7.02	0.0427
20	3	-1	-1	-1	-1	-1	-1	-1	-1	-1	-1	-1	-1	-1	-1	-1	-1	-1	-1	-1	6.93059	6.88	0.0464
10	4	-1	1	-1	-1	-1	-1	1	1	-1	1	1	-1	-1	1	1	1	1	-1	1	10.0017	9.96	0.0427
5	5	-1	1	1	-1	1	1	-1	-1	1	1	1	1	-1	1	-1	1	-1	-1	-1	3.70862	3.70	0.0096

18	6	-1	-1	1	1	1	1	-1	1	-1	1	-1	-1	-1	-1	1	1	-1	1	1	4.81782	4.83	-0.0096
16	7	1	1	1	1	-1	1	-1	1	-1	-1	-1	-1	1	1	-1	1	1	-1	-1	11.1397	11.15	-0.0058
7	8	-1	-1	-1	1	1	-1	1	1	-1	-1	1	1	1	1	-1	1	-1	1	-1	7.4223	7.46	-0.0427
15	9	1	1	1	-1	1	-1	1	-1	-1	-1	-1	1	1	-1	1	1	-1	-1	1	7.37184	7.37	0.0058
1	10	1	1	-1	-1	1	1	1	1	-1	1	-1	1	-1	-1	-1	-1	1	1	-1	8.11667	8.11	0.0096
2	11	-1	1	1	-1	-1	1	1	1	1	-1	1	-1	1	-1	-1	-1	-1	1	1	5.13966	5.10	0.0427
19	12	1	-1	-1	1	1	1	1	-1	1	-1	1	-1	-1	-1	-1	1	1	-1	1	3.09943	3.11	-0.0096
8	13	-1	-1	-1	-1	1	1	-1	1	1	-1	-1	1	1	1	1	-1	1	-1	1	10.8178	10.81	0.0058

6	14	-1	-1	1	1	-1	1	1	-1	-1	1	1	1	1	-1	1	-1	1	-1	-1	4.77759	4.82	-0.0427
4	15	1	1	-1	1	1	-1	-1	1	1	1	1	-1	1	-1	1	-1	-1	-1	-1	7.30632	7.31	-0.0058
17	16	-1	1	1	1	1	-1	1	-1	1	-1	-1	-1	-1	1	1	-1	1	1	-1	9.50172	9.51	-0.0058
3	17	1	-1	1	1	-1	-1	1	1	1	1	-1	1	-1	1	-1	-1	-1	-1	1	8.13851	8.18	-0.0464
12	18	-1	1	-1	1	-1	-1	-1	-1	1	1	-1	1	1	-1	-1	1	1	1	1	12.5245	12.57	-0.0464
11	19	1	-1	1	-1	-1	-1	-1	1	1	-1	1	1	-1	-1	1	1	1	1	-1	5.92701	5.92	0.0096
14	20	1	1	-1	1	-1	1	-1	-1	-1	-1	1	1	-1	1	1	-1	-1	1	1	5.37759	5.42	-0.0464
Variable level		g/L	g/L	g/L	g/L	g/L	g/L	g/L	g/L	mL	g/L	(°C)	(pH)	mL	%	rpm	days	days	-	-			

-1	0.7	0.5	0.02	0.04	0.016	0.003	0.003	0.0005	0.5	0	25	7	400	3	0	7	20					
1	3.4	3	0.06	0.1	0.056	0.009	0.009	0.001	1.5	10	33	10	600	6	120	20	30					
